# Performance of Bleeding Risk Scores for Major Bleeding in Anticoagulated Patients with Pulmonary Embolism: Insights from the CURES Registry-2

**DOI:** 10.1055/a-2642-0241

**Published:** 2025-07-17

**Authors:** Yuzhi Tao, Hong Chen, Chunling Dong, Jie Zhang, Yiwei Shi, Xiaomao Xu, Maoyun Wang, Ling Zhu, Juhong Shi, Yingqun Ji, Hong Chen, Zhe Cheng, Yongjun Tang, Yanxia Li, Chaosheng Deng, Qin Luo, Pinyao Lu, Yuanhua Yang, Linfeng Xi, Yu Zhang, Rui Liang, Dingyi Wang, Guohui Fan, Wanmu Xie, Jun Wan, Zhu Zhang, Shuai Zhang, Yunxia Zhang, Qiang Huang, Qian Gao, Min Liu, Peiran Yang, Shengfeng Wang, Chen Wang, Zhenguo Zhai, on behalf of the CURES investigators

**Affiliations:** 1 Department of Pulmonary and Critical Care Medicine, The First Bethune Hospital of Jilin University, Jilin University, Changchun, China; 2 National Centre for Respiratory Medicine, State Key Laboratory of Respiratory Health and Multimorbidity, National Clinical Research Centre for Respiratory Diseases, Beijing, China; 3 Institute of Respiratory Medicine, Chinese Academy of Medical Sciences, Beijing, China; 4 Department of Pulmonary and Critical Care Medicine, China-Japan Friendship Hospital, Beijing, China; 5 Department of Pulmonary and Critical Care Medicine, The First Affiliated Hospital of Chongqing Medical University, Chongqing, China; 6 Department of Pulmonary and Critical Care Medicine, The Second Hospital, Jilin University, Changchun, China; 7 Department of Pulmonary and Critical Care Medicine, The First Hospital of Shanxi Medical University, Taiyuan, China; 8 Department of Pulmonary and Critical Care Medicine, Beijing Hospital, Beijing, China; 9 Department of Pulmonary and Critical Care Medicine, West China Hospital of Sichuan University, Chengdu, China; 10 Department of Pulmonary and Critical Care Medicine, Shandong Provincial Hospital Affiliated to Shandong First Medical University, Jinan, China; 11 Department of Pulmonary and Critical Care Medicine, Peking Union Medical College Hospital, Beijing, China; 12 Department of Pulmonary and Critical Care Medicine, Shanghai East Hospital, Shanghai, China; 13 Department of Pulmonary and Critical Care Medicine, The Second Affiliated Hospital of Harbin Medical University, Harbin, China; 14 Department of Pulmonary and Critical Care Medicine, The First Affiliated Hospital of Zhengzhou University, Zhengzhou, China; 15 Department of Pulmonary and Critical Care Medicine, Xiangya Hospital Central South University, Changsha, China; 16 Department of Pulmonary and Critical Care Medicine, The First Affiliated Hospital of Dalian Medical University, Dalian, China; 17 Department of Pulmonary and Critical Care Medicine, The first affiliated Hospital of Fujian Medical University, Fuzhou, China; 18 Department of Pulmonary and Critical Care Medicine, The Third Clinical Medical College of Xinjiang Medical University, Urumqi, China; 19 Department of Pulmonary and Critical Care Medicine, Affiliated Hospital of Chengde Medical university, Chengde, China; 20 Department of Pulmonary and Critical Care Medicine, Beijing Chao-Yang Hospital, Capital Medical University, Beijing, China; 21 Department of Pulmonary and Critical Care Medicine, China-Japan Friendship Hospital, Capital Medical University, Beijing, China; 22 Beijing University of Chinese Medicine China-Japan Friendship School of Clinical Medicine, Beijing, China; 23 Department of Pulmonary and Critical Care Medicine, Beijing Anzhen Hospital, Capital Medical University, Beijing, China; 24 Department of Radiology, China-Japan Friendship Hospital, Beijing, China; 25 Department of Physiology, State Key Laboratory of Medical Molecular Biology, Institute of Basic Medical Sciences, Chinese Academy of Medical Sciences, School of Basic Medicine Peking Union Medical College, Beijing, China; 26 Department of Epidemiology and Biostatistics, School of Public Health, Peking University, Beijing, China; 27 Key Laboratory of Epidemiology of Major Diseases (Peking University), Ministry of Education, Beijing, China

**Keywords:** pulmonary embolism, anticoagulation, major bleeding, score, prediction

## Abstract

**Background:**

Most bleeding risk scores for pulmonary embolism (PE) were developed in patients receiving traditional anticoagulants. Evidence in East Asian populations and its applicability to direct oral anticoagulants (DOACs) remain limited.

**Methods:**

This post-hoc analysis was based on a multicentre, prospective study (NCT02943343) conducted from 2016 to 2021. The predictive performance of bleeding risk scores was assessed using a time-dependent area under the receiver operating characteristic curve (AUC), net reclassification improvement (NRI), and decision curve analysis (DCA). Propensity score matching (PSM) was adjusted for baseline characteristics. We analyzed the impact of initial DOAC versus low-molecular-weight heparin (LMWH) on outcomes. The endpoint was major bleeding (MB) within 90 days and composite outcomes (all-cause mortality, recurrent VTE, and MB).

**Results:**

Of 7,619 patients with PE, 1.4% (107 patients) experienced MB within 90 days. The RIETE score showed a modest predictive ability (AUC: 0.70; 95% CI, 0.65–0.75) for predicting 90-day MB and demonstrated a predictive advantage in the DCA results. NRI also revealed significantly better reclassification capability than the other scores, except for HAS-BLED. Among low-risk patients classified by the RIETE score, initial DOAC treatment significantly reduced 14-day composite outcomes compared with LMWH (HR = 0.13; 95% CI, 0.02–0.93). Furthermore, DOACs at discharge did not increase the risk of MB or composite outcomes.

**Conclusion:**

RIETE score shows modest performance in predicting MB and identifying low bleeding risk in PE patients, which could potentially guide early DOAC use. Further studies are needed to test its clinical utility, especially in East Asian populations.

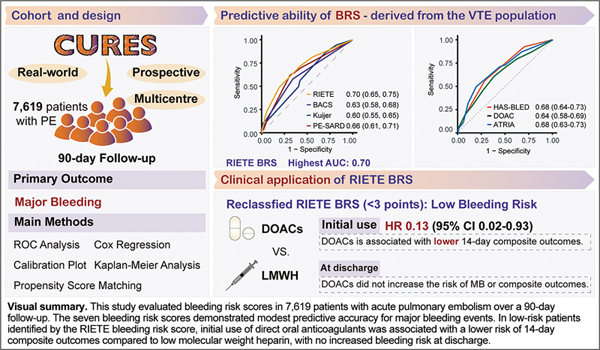

## Introduction

Pulmonary embolism (PE) is one of the major causes of cardiovascular morbidity and mortality, significantly contributing to global healthcare burdens. 1 Despite advances in anticoagulation and thrombolysis, managing PE remains challenging because of the associated risk of major bleeding (MB). 2 Identifying risk factors for MB and developing individualized treatment strategies are crucial to improving patient outcomes.

The Kuijer score has demonstrated good clinical utility in predicting MB and mortality in patients with venous thromboembolism (VTE). 3 4 The BACS (Bleeding, Age, Cancer, Syncope) score, developed to assess bleeding risk in PE patients undergoing systemic thrombolysis, has been externally validated using the COMMAND VTE (Contemporary Management and Outcomes in VTE) dataset. 5 The PE-SARD score (Syncope, Anemia, Renal Dysfunction) was designed to predict early MB risk in patients with PE. 6 Although simple and clinically applicable, 7 the PE-SARD score has shown suboptimal performance during external validation, especially in older patients. 8 The RIETE (Registro Informatizado de Enfermedad TromboEmbólica) bleeding risk score (RIETE BRS) effectively identifies bleeding risk in patients with PE; however, caution is advised when applying it to subgroups such as cancer patients and the elderly. 9 10 The HAS-BLED score has been widely validated in atrial fibrillation (AF) patients, helping to guide anticoagulant therapy, reduce MB risks, and improve treatment outcomes. 11 12 13 14 It is one of the most reliable tools for predicting bleeding events, including intracranial haemorrhage (ICH). 15 Additionally, it has shown potential in predicting bleeding risk in VTE populations. 16 Similarly, the DOAC 17 and ATRIA scores, 18 developed for AF patients, effectively predict MB events. However, their predictive performance in the VTE populations of China requires further prospective validation.

Individualized management based on bleeding risk assessment remains a critical area of study. Despite advances in bleeding risk scores for PE, their integration into clinical practice is still limited. Most of these scores were developed and validated in Western populations with different genetics, treatment doses, and comorbidities. The “East Asian Paradox” has been proposed by scholars, highlighting the unique balance of thrombosis and bleeding risks observed in East Asian populations compared with Western populations during antithrombotic treatment, underscoring the need for tailored antithrombotic strategies for East Asians. 19 Varying clinical settings and patient-specific differences further influence the accuracy of existing scores. Notably, these bleeding risk scores have not been prospectively tested in large Chinese cohorts of PE patients. Furthermore, Chinese guidelines for managing PE bleeding risk still rely on the 2012 ACCP guidelines, highlighting the need to test these scores and develop tools tailored to Chinese populations.

This study aims to evaluate the performance of four VTE-related bleeding risk scores in predicting MB associated with anticoagulation within 90 days of PE diagnosis and to determine whether a bleeding risk score with better predictive performance can effectively guide the selection of appropriate initial anticoagulants. The study will also explore the predictive value of bleeding risk scores derived from the AF population in patients with PE.

## Methods

### Study Design

The China pUlmonary thromboembolism REgistry Study (CURES) is a multicentre, prospective, observational study of consecutive patients with PE (NCT02943343). The study followed the Declaration of Helsinki and was approved by institutional review boards and ethics committees (approval No. 2016-SSW-7). Informed consent was obtained from all patients or their legal representatives. Patients were enrolled from 1 January 2016 to 31 December 2021, with at least 90 days of follow-up. Adults with newly confirmed PE were included, while those with significant baseline missing data or who refused long-term follow-up were excluded. This study adhered to the Transparent Reporting of a Multivariable Prediction Model for Individual Prognosis or Diagnosis (TRIPOD) statement ( Supplementary Method S1 , available in the online version).

### Data Collection and Study Endpoints

Data were prospectively collected for all patients, covering demographic characteristics, clinical presentation, comorbidities, risk factors, laboratory findings, acute-phase treatment details, and outcomes. For the missing data variables, including labile INR, alcohol use, antiplatelet use, and nonsteroidal anti-inflammatory drug (NSAID) use, we assigned a default value of 0 for these variables. The primary outcome was the occurrence of MB within 90 days of diagnosis, defined according to the International Society on Thrombosis and Haemostasis (ISTH) criteria. The composite outcome was all-cause mortality, recurrent VTE, or MB ( Supplementary Method S2 , available in the online version). A central adjudication committee reviewed all suspected events based on original medical documentation. Outcome data were collected during follow-up through in-person assessments, telephone interviews, and regular examinations of inpatient medical records. If patients experienced multiple events, only the first MB was recorded.

### Statistical Analysis

Baseline characteristics of categorical variables were expressed as percentages. The proportion of missing data are detailed in Supplementary Method S3 (available in the online version), with missing values imputed using multiple imputations. The discriminative performance of the PE-SARD, BACS, Kuijer, RIETE, HAS-BLED, ATRIA, and DOAC scores was evaluated using a time-dependent area under the receiver operating characteristic (ROC) curve (AUC) analysis. Sensitivity, specificity, positive and negative predictive values, and likelihood ratios (LRs) were calculated for each score. Risk reclassification was assessed using continuous net reclassification improvement (NRI) and integrated discrimination improvement (IDI), accounting for competing risks. Decision curve analysis (DCA) was used to evaluate the clinical usefulness of the prediction scores. 20

We performed a calibration plot and conducted the Hosmer-Lemeshow goodness-of-fit test to evaluate the calibration performance of the seven bleeding scores for MB. A Cox regression model was applied to identify risk factors for MB in the presence of competing risks. Factors with a *P* -value <0.1 or those deemed clinically relevant were included in the multivariate analysis. The risks of high- and intermediate-risk groups were compared with the low-risk group across the four scores using unadjusted sub-hazard ratios (sHRs) with 95% confidence intervals (CIs). Kaplan-Meier curves were used to assess the cumulative incidence of MB among patients classified as low, intermediate, and high risk. The Fine-Gray subdistribution hazard model was applied to estimate cumulative incidence, accounting for all-cause mortality as a competing event. Propensity score matching (PSM) was employed to minimize confounding influences. For the initial anticoagulant choice, patients in the DOACs and LMWH groups were matched at a 1:4 ratio (random sampling method; clamp value of 0.05) based on factors associated with MB and mortality risk. At discharge, the anticoagulant groups were compared in a 1:1 ratio (random sampling method; clamp value of 0.001). The balance between the two groups after matching was assessed using standardized mean differences (SMD). Group comparisons of rates were then performed using either Fisher's exact test or the chi-square test.

Patients lost to follow-up were censored at the last visit. Sensitivity analyses were conducted to assess the impact of imputation on model predictions using both imputed and original datasets and to assess the effect of excluding patients lost to follow-up. Bonferroni correction was applied for pairwise comparisons between groups to adjust *P* -values. A two-sided *P* -value <0 0.05 was considered statistically significant. All analyses were performed using R software version 4.0.2.

## Results

### Patient Characteristics

A total of 7,619 patients with acute PE were included in the analysis ( Supplementary Fig. S1 , available in the online version). The mean age was 63, with 3,987 men (52.3%), and the mean BMI was 24.2 kg/m ^2^ . Overall, 1,098 patients (14.4%) had cancer, and 112 (1.5%) had a history of MB. Syncope as presentation of PE was recorded in 585 patients (7.7%), 751 (9.9%) had a pulse rate of ≥110 beats/min, and 1,635 (21.5%) showed signs of right ventricular dysfunction (RVD). At admission, 3,025 patients (39.7%) had anaemia, and 898 (11.8%) had renal dysfunction. During the acute treatment phase, 6,052 patients (79.4%) received LMWH, 652 (8.6%) were treated with DOACs, and 392 (5.1%) underwent systemic thrombolysis ( Table 1 ). Of all participants, 412 (5.4%) were lost to follow-up, and 521 (6.8%) died within 90 days. The MB rate was 1.4% (107 patients), and 35 (0.5%) experienced recurrent VTE. We also compared the baseline characteristics with those from other cohorts ( Supplementary Tables S1 and S2 , available in the online version).

**Table 1:** Baseline characteristics of patients with pulmonary embolism in CURES

Characteristics	All ( *N* = 7,619)
**Clinical characteristics**	
Age, mean ± sd (y)	63 ± 15
Age >75, *n* (%)	1,737 (22.8)
Sex, male, *n* (%)	3,987 (52.3)
Body mass index (kg/m ^2^ )	24.2 ± 3.8
Underweight (BMI <18.5)	377 (4.9)
**Risk factors and comorbid diseases,** ***n*** **(%)**	
History of VTE	1840 (24.2)
Cancer	1,098 (14.4)
Active cancer	799 (10.5)
Recent surgery a	906 (11.9)
Immobility ≥3 days	621 (8.2)
Chronic lung disease	1,044 (13.7)
Congestive heart failure	476 (6.2)
Previous stroke	750 (9.8)
Stroke/transient ischaemic attack/embolism history	1,739 (22.8)
Hypertension	2,749 (36.1)
Uncontrolled hypertension	408 (5.4)
Diabetes	923 (12.1)
History of bleeding a	362 (4.8)
Recent major bleeding a	112 (1.5)
**Clinical symptoms and signs at presentation,** ***n*** **(%)**	
Systolic blood pressure (mmHg)	127.8 ± 19.6
Syncope b	585 (7.7)
Pulse ≥110 beats/min	751 (9.9)
Right ventricular dysfunction	1,635 (21.5)
Simplified PESI score ≥1	4,820 (63.3)
**ESC-defined risk categories,** ***n*** **(%)**	
High risk	450 (5.9)
Intermediate-high risk	1,137 (14.9)
Intermediate-low risk	4,310 (56.6)
Low risk	1,722 (22.6)
**Laboratory findings,** ***n*** **(%)**	
Anaemia	3,025 (39.7)
Platelet count <100 × 10 ^9^ ·L ^−1^	364 (4.8)
Positive troponin c	1,545 (20.3)
Liver dysfunction	156 (2.0)
Renal dysfunction d	898 (11.8)
eGFR 30–59	806 (10.6)
eGFR <30	92 (1.2)
Serum creatinine, mean ± sd	74.3 ± 33.1
Creatinine levels >1.2 mg/dL	633 (8.3)
**Treatment in the acute phase,** ***n*** **(%)**	
LMWH	6,052 (79.4)
Unfractioned heparin	353 (4.6)
DOACs	652 (8.6)
Vitamin K antagonist	163 (2.1)
Thrombolysis e	392 (5.1)
Inferior vena cava filter use	16 (0.2)
Other/unknown	115 (1.5)
No anticoagulation	284 (3.7)
**Anticoagulation of discharge,** ***n*** **(%)**	
LMWH	1,088 (14.3)
DOACs	3,507 (46.0)
Vitamin K antagonist	2,501 (32.8)
Other/unknown	202 (2.7)
No anticoagulation	321 (4.2)
**Outcome, 90 days,** ***n*** **(%)**	
Major bleeding	107 (1.4)
Clinically relevant non-major bleeding	421 (5.5)
Recurrent VTE	35 (0.5)
Overall death	521 (6.8)

Abbreviations: COPD, chronic obstructive pulmonary disease; CURES, China pUlmonary thromboembolism REgistry Study; DOACs, direct oral anticoagulants; DVT, deep venous thrombosis; eGFR, estimated glomerular filtration rate; ESC, European Society of Cardiology; LMWH, low-molecular-weight heparin; *N* , number; PE, pulmonary embolism; PESI, Pulmonary Embolism Severity Index; SD, standard deviation; VTE, venous thromboembolism.

a Within the past 3 months.

b Self-reported by the patient (or witnesses).

c Troponin I >0.4 or troponin T >1.

d Renal dysfunction was defined by eGFR values of <60 mL/min/1.73 m ^2^ (the eGFR values were based on the simplified Chronic Kidney Disease Epidemiology Collaboration (eGFR _CKD-EPI_ ) formula).

e Systemic thrombolysis, with most patients receiving anticoagulation therapy prior to thrombolysis.

### Risk Stratification Based on Scores Derived from VTE Populations

**Fig. 1:**
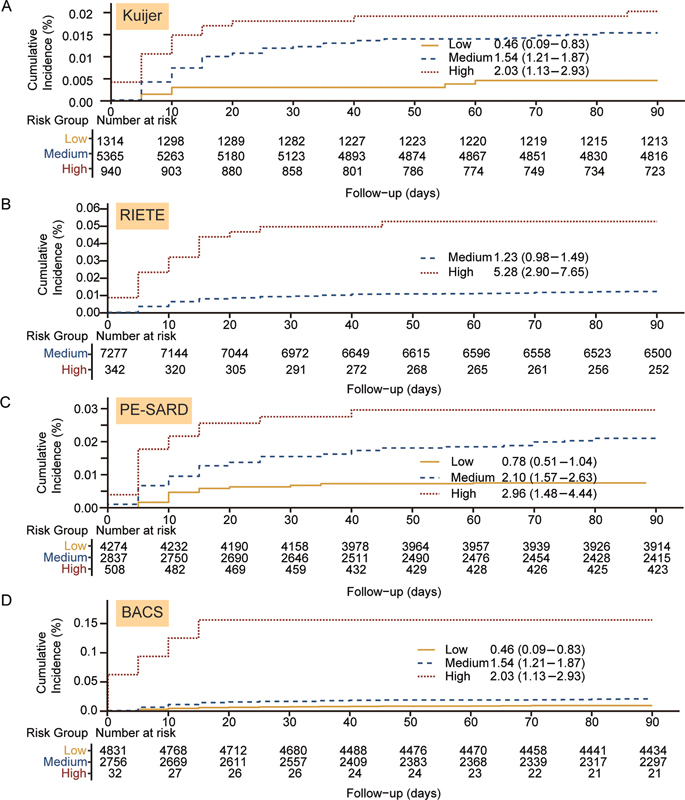
Kaplan-Meier curve showing cumulative incidence of major bleeding at 90 days by four bleeding risk scores. ( **A** ) Kuijer score: low-risk group: 0.46% (95% CI, 0.09–0.83%), intermediate-risk group: 1.54% (95% CI, 1.21–1.87%), high-risk groups: 2.03% (95% CI, 1.13–2.93%). ( **B** ) RIETE score: intermediate-risk group: 1.23% (95% CI, 0.98–1.49%), high-risk groups: 5.28% (95% CI, 2.90–7.65%). ( **C** ) PE-SARD score: low-risk group: 0.78% (95% CI, 0.51–1.04%), intermediate-risk group: 2.10% (95% CI, 1.57–2.63%), high-risk groups: 2.96% (95% CI, 1.48–4.44%). ( **D** ) BACS score: low-risk group: 0.46% (95% CI, 0.09–0.83%), intermediate-risk group: 1.54% (95% CI, 1.21–1.87%), high-risk groups: 2.03% (95% CI, 1.13–2.93%).

The RIETE, Kuijer, PE-SARD, and BACS scores classified 342 (4.5%), 940 (12.3%), 508 (6.7%), and 32 (0.4%) patients as high risk, respectively; 7,277 (95.5%), 5,365 (70.4%), 2,837 (37.2%), and 2,756 (36.2%) patients as intermediate risk; and 0, 1,314 (17.2%), 4,274 (56.1%), and 4,831 (63.4%) patients as low risk. The RIETE BRS did not classify any patient as low risk, as every patient was assigned 1 point for diagnosing PE ( Table 2 ). The predicted bleeding rates were similar to observed rates across all four scores ( Supplementary Fig. S2 , available in the online version). The cumulative incidence of MB increased with higher risk stratification levels across all four scores (adjusted *P* -value <0.05). However, no significant difference was observed between intermediate- and high-risk patients classified using the Kuijer and PE-SARD scores (adjusted *P* -value >0.1; Fig. 1 ).

**Table 2:** The cumulative incidence of major bleeding in low-, intermediate-, and high-risk groups, according to the bleeding risk scores

Scores	Low risk		Intermediate risk		High risk	
	*n* / *N* a	% (95% CI)	*n* / *N* a	% (95% CI)	*n* / *N* a	% (95% CI)
RIETE	NA		89/7,277	1.23 (0.98–1.49)	18/342	5.28 (2.90–7.65)
Kuijer	6/1,314	0.46 (0.09–0.83)	82/5,365	1.54 (1.21–1.87)	19/940	2.03 (1.13–2.93)
PE-SARD	33/4,274	0.78 (0.51–1.04)	59/2,837	2.10 (1.57–2.63)	15/508	2.96 (1.48–4.44)
BACS	45/4,831	0.94 (0.67–1.21)	57/2,756	2.08 (1.55–2.62)	5/32	15.63 (2.84–28.41)

a Number of major bleeding ( *n* ) and overall number of participants in risk group ( *N* ).

### Performance of Bleeding Risk Scores Derived from VTE Populations

**Fig. 2:**
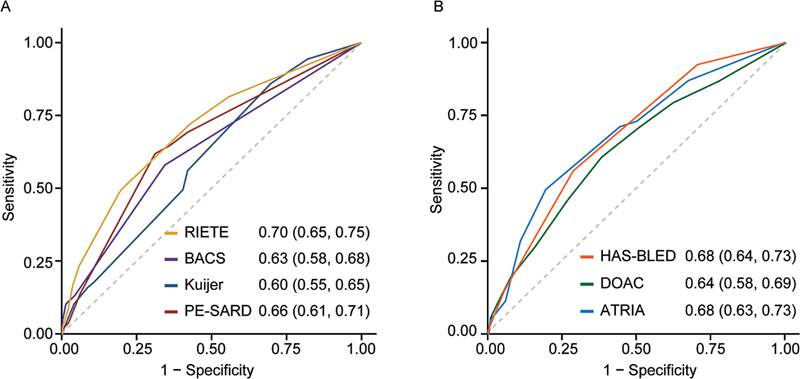
Receiver operating characteristic curves (AUCs) for each bleeding risk score. ( **A** ) AUCs for 90-day major bleeding according to RIETE (0.70, 95% CI, 0.65–0.75), BACS (0.63, 95% CI, 0.58–0.68), Kuijer (0.60, 95% CI, 0.55–0.65), and PE-SARD (0.66, 95% CI, 0.61–0.71) scores. ( **B** ) AUCs for 90-day major bleeding according to HAS-BLED (0.68, 95% CI, 0.64–0.73), DOAC (0.64, 95% CI, 0.58–0.69), ATRIA (0.68, 95% CI, 0.63–0.73).

No significant collinearity was found between thrombolysis, 2019 European Society of Cardiology (ESC) risk stratification, and bleeding risk scores ( Supplementary Table S3 , available in the online version). Patients classified as high risk by the RIETE BRS had a significantly higher MB risk compared with those at intermediate risk (90 days, adjusted sHR: 4.11, 95% CI, 2.47–6.87; *P* -value <0.05), with similar findings at 14-day and 30-day follow-ups. For the other scores, patients at high or intermediate risk had a significantly higher MB risk than those at low risk ( Supplementary Table S4 , available in the online version). During the 90-day follow-up, all scores had AUCs between 0.6 and 0.7, with the RIETE BRS having the highest AUC (0.70, 95% CI, 0.65–0.75) and the Kuijer score having the lowest (0.60, 95% CI, 0.55–0.65) ( Table 3 , Fig. 2 , Supplementary Table S5 , and Supplementary Fig. S3 , available in the online version). The RIETE, PE-SARD, Kuijer, and BACS scores showed similar Brier scores, with acceptable calibration for all except the BACS score in predicting MB at 90 days ( Table 3 , Supplementary Fig. S4 , available in the online version).

**Table 3:** Predictive performance of bleeding risk scores for major bleeding events

Scores	Outcome	Brier score	AUC (95% CI)	Hosmer-Lemeshow	
				*P* -value	*χ* ^2^
RIETE	MB within 14 days	0.0083	0.69 (0.62–0.75)	0.41	2.90
	MB within 30 days	0.0087	0.69 (0.63–0.75)	0.34	3.33
	MB within 90 days	0.0101	0.70 (0.65–0.75)	0.43	2.74
PE-SARD	MB within 14 days	0.0042	0.64 (0.58–0.71)	0.72	1.32
	MB within 30 days	0.0042	0.65 (0.59–0.70)	0.73	1.30
	MB within 90 days	0.0059	0.66 (0.61–0.71)	0.26	3.97
Kuijer	MB within 14 days	0.0017	0.60 (0.54–0.66)	0.48	3.50
	MB within 30 days	0.0017	0.60 (0.55–0.66)	0.37	4.29
	MB within 90 days	0.0018	0.60 (0.55–0.65)	0.32	4.71
BACS	MB within 14 days	0.0063	0.63 (0.57–0.70)	0.17	1.91
	MB within 30 days	0.0074	0.63 (0.58–0.69)	0.07	3.17
	MB within 90 days	0.0072	0.63 (0.58–0.68)	0.04	4.42
ATRIA	MB within 14 days	0.0045	0.67 (0.61–0.74)	0.004	8.44
	MB within 30 days	0.0045	0.68 (0.62–0.73)	0.007	7.22
	MB within 90 days	0.0062	0.68 (0.63–0.73)	0.03	4.59
HAS-BLED	MB within 14 days	0.0058	0.67 (0.61–0.73)	0.52	0.47
	MB within 30 days	0.0074	0.68 (0.63–0.73)	0.49	0.48
	MB within 90 days	0.0077	0.68 (0.64–0.73)	1.43	0.23
DOAC	MB within 14 days	0.0036	0.65 (0.58–0.72)	0.97	1.70
	MB within 30 days	0.0045	0.65 (0.59–0.71)	0.87	3.17
	MB within 90 days	0.0045	0.64 (0.58–0.69)	0.86	3.30

Abbreviations: DOAC, direct oral anticoagulant; MB, major bleeding.

### Comparison of Bleeding Risk Scores Derived from VTE and AF Populations

HAS-BLED, ATRIA, and DOAC scores, originally designed for AF, were compared with VTE-derived scores in this study. Throughout the follow-up, their AUCs ranged from 0.6 to 0.7. All three scores showed similar Brier scores, with only HAS-BLED and DOAC demonstrating acceptable calibration ( Table 3 , Fig. 2 , Supplementary Table S6 , Supplementary Figs. S3 and S5 , available in the online version). Moreover, at the 90-day follow-up, the RIETE BRS showed a significantly higher overall NRI and negative NRI than the other scores, except compared with the HAS-BLED score. Specifically, the RIETE BRS correctly reclassified 12.37% of patients into the high-risk group and incorrectly reclassified 18.98% into the low-risk group compared with HAS-BLED. Although RIETE BRS showed an improvement in IDI, the improvement did not reach statistical significance when compared with HAS-BLED and BACS scores ( Table 4 ). The RIETE BRS demonstrated a higher net benefit across a broad range of threshold probabilities than all other scores except the BACS score. The RIETE BRS outperformed the BACS score in the 0 to 3%, while the BACS score was superior in the 3 to 10% range ( Supplementary Fig. S6 , available in the online version). Similar results were observed at the 14-day and 30-day follow-ups.

**Table 4:** Comparison of NRI and IDI for other bleeding scores relative to the RIETE bleeding risk score

Score	Follow-up days	NRI % (95% CI)			IDI % (95% CI)
		Positive	Negative	Overall	
PE-SARD	14	16.67 (−4.69, 40.91)	26.02 (23.75, 28.25)	42.68 (21.01, 67.07)	0.36 (0.09, 0.6)
	30	10.34 (−11.38, 29.83)	27.04 (24.73, 29.20)	37.39 (16.06, 56.83)	0.38 (0.13, 0.61)
	90	4.67 (−14.77, 23.81)	32.32 (1.11, 30.13)	36.99 (17.81, 56.71)	0.36 (0.10, 0.61)
Kuijer	14	22.22 (0, 46.47)	29.79 (27.64, 31.91)	52.02 (29.96, 75.67)	0.56 (0.26, 0.84)
	30	19.54 (−2.00, 39.88)	31.01 (28.72, 33.37)	50.55 (29.46, 70.90)	0.58 (0.30, 0.85)
	90	19.63 (1.96, 39.63)	32.35 (30.08, 34.44)	51.97 (34.33, 71.87)	0.69 (0.40, 0.98)
BACS	14	11.11 (−12.70, 34.43)	18.37 (16.14, 20.63)	29.48 (6.21, 52.82)	0.01 (−0.37, 0.34)
	30	10.34 (−10.56, 32.22)	19.42 (17.37, 21.73)	29.76 (7.95, 51.54)	−0.07 (−0.46, 0.28)
	90	17.76 (0, 36.23)	21.09 (18.74, 23.41)	38.85 (20.06, 57.85)	0.09 (−0.24, 0.41)
ATRIA	14	−10.74 (−33.43, 12.18)	58.06 (56.21, 59.81)	47.32 (24.24, 70.35)	0.32 (0.11, 0.58)
	30	−12.17 (−33.82, 9.76)	58.14 (56.27, 59.94)	45.96 (24.41, 67.97)	0.35 (0.15, 0.58)
	90	−17.24 (−35.60, 1.33)	58.20 (56.36, 60.00)	40.96 (21.97, 59.76)	0.33 (0.11, 0.58)
HAS-BLED	14	19.47 (−5.33, 41.44)	−19.91 (−22.01, −17.70)	−0.44 (−25.31, 21.74)	0.16 (−0.15, 0.48)
	30	19.36 (−2.69, 39.31)	−34.68 (−36.71, −32.56)	−15.31 (−37.62, 4.66)	0.05 (−0.27, 0.41)
	90	12.37 (−6.05, 31.44)	−18.98 (−21.32, −16.83)	−6.62 (−25.56, 12.10)	0.15 (−0.15, 0.48)
DOAC	14	11.45 (−12.23, 35.49)	23.90 (21.60, 25.96)	35.35 (11.63, 58.54)	0.39 (0.15, 0.65)
	30	6.10 (−14.55, 28.04)	23.91 (21.71, 26.13)	30.01 (9.13, 52.21)	0.36 (0.11, 0.60)
	90	11.00 (−8.44, 30.20)	23.96 (21.82, 26.21)	34.96 (15.78, 53.97)	0.49 (0.24, 0.73)

Abbreviations: IDI, integrated discrimination improvement; NRI, net reclassification improvement.

### Predictors of Major Bleeding

The results of the univariable analysis for potential predictors are shown in Supplementary Fig. S7 (available in the online version). Multivariate analysis indicated that age ≥65 years (sHR = 1.61, 95% CI, 1.06–2.47), active cancer (sHR = 1.68, 95% CI, 1.02–2.74), gastrointestinal diseases (sHR = 2.04, 95% CI, 1.17–3.55), prior MB (sHR = 5.75, 95% CI, 2.97–11.15), pulse >100 beats/min (sHR = 1.61, 95% CI, 1.07–2.43), anaemia (sHR = 2.49, 95% CI, 1.61–3.83), PLT <100 × 10 ^9^ /L (sHR = 2.72, 95% CI, 1.55–4.76), and CR >1.2 mg/dL (sHR = 1.77, 95% CI, 1.02–3.07) were independent predictors of MB at 90 days ( Supplementary Fig. S8 , available in the online version). Similar results were observed in the subgroup of patients receiving anticoagulation. Additionally, connective tissue disease (CTD, sHR = 2.46, 95% CI, 1.09–5.53) and neurological disease (sHR = 1.95, 95% CI, 1.13–3.38) were also predictors of MB in anticoagulated patients ( Supplementary Figs. S9 and S10 , available in the online version).

### Subgroup Analyses

In a subgroup analysis of 6,921 anticoagulated patients, the AUCs for predicting MB at 90 days were as follows: RIETE BRS, 0.71 (95% CI, 0.66–0.77); Kuijer score, 0.58 (95% CI, 0.53–0.64); PE-SARD score, 0.65 (95% CI, 0.60–0.71); BACS score, 0.62 (95% CI, 0.56–0.68); HAS-BLED score, 0.70 (95% CI, 0.65–0.75); ATRIA score, 0.70 (95% CI, 0.65–0.76) and DOAC score, 0.64 (95% CI, 0.58–0.70) ( Supplementary Table S7 , available in the online version). Among the scores that effectively predict MB, the RIETE BRS also exhibited the highest sensitivity (75.35%, 95% CI, 64.75–84.01) and Negative Predictive Value (NPV) (99.47%, 95% CI, 99.26–99.62). The NRI and IDI results were generally consistent with those observed in the overall population. Compared with the Kuijer, PE-SARD, BACS, ATRIA, and DOAC scores, the RIETE BRS showed superior NRIs of 60.70, 48.60, 45.10, 41.08, and 42.77%, respectively. Compared with HAS-BLED, the RIETE BRS showed a decrease in NRI of 11.45%, but this difference was not statistically significant. Similar results were observed at other follow-up intervals ( Supplementary Table S8 , available in the online version). In non-cancer patients, AUCs ranged from 0.57 to 0.72. In cancer patients, performance was generally poor, with AUCs ranging from 0.44 to 0.63 ( Supplementary Tables S9 and S10 , available in the online version).

### Sensitivity Analyses

We also performed a sensitivity analysis based on scores derived from the VTE population. After excluding patients with missing values, the four bleeding scores showed moderate discriminative performance for MB at 14, 30, and 90 days, with AUCs ranging from 0.6 to 0.7. The RIETE BRS had the highest AUC at 90 days (0.70, 95% CI, 0.65–0.75), consistent with the primary findings. Excluding patients lost to follow-up within 90 days did not affect the scores' discriminative performance. A similar analysis in the anticoagulated subgroup showed results consistent with the primary findings ( Supplementary Table S11 , available in the online version).

### Clinical Application

Patients with PE who received anticoagulation and subsequently experienced MB had significantly worse 90-day outcomes, including a higher risk of death (adjusted HR = 4.12, 95% CI, 2.78–6.11) and composite events (adjusted HR = 4.34, 95% CI, 2.93–6.42) ( Supplementary Table S12 , Supplementary Fig. S11 , available in the online version). To assess the impact of initial anticoagulant choice, we analyzed outcomes in patients receiving either LMWH or DOACs without thrombolysis. For patients where data were available, the initial doses were administered according to guidelines, with dosage adjustments during treatment based on the physician's clinical judgement.

**Fig. 3:**
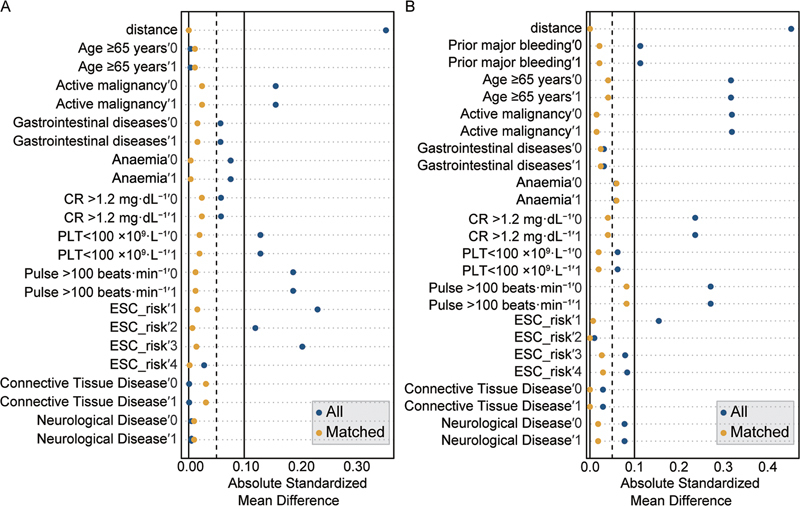
Assessing the quality of matches between direct oral anticoagulant (DOAC) and low-molecular-weight heparin (LMWH) groups during the acute phase of initial anticoagulation. ( **A** ) Standardized mean difference (SMD) before and after propensity score matching (PSM) in patients classified as low bleeding risk according to the RIETE BRS. ( **B** ) SMD before and after PSM in patients classified as high bleeding risk according to the RIETE BRS. CR, creatinine; ESC, European Society of Cardiology; PLT, platelet.

The optimal threshold of the RIETE BRS was used to categorize patients into low- and high-risk groups, with PSM balancing covariates between the two anticoagulant groups ( Supplementary Table S13 [available in the online version], Fig. 3 ). Among low-risk patients, initial use of DOACs was associated with a lower incidence of composite outcomes within 14 days compared with LMWH (HR = 0.13, 95% CI, 0.02–0.93, Supplementary Tables S14 and S15 , available in the online version). In high-risk patients, the initial anticoagulant choice did not significantly affect outcomes ( Supplementary Table S16 , available in the online version). Considering that patients may switch anticoagulants at discharge, we excluded those who died in hospital and categorized the remaining patients into LMWH and DOAC groups based on discharge medication. After adjusting for confounding factors using PSM, patients in the low-risk group discharged on sequential DOACs did not have a higher risk of MB or composite outcomes than LMWH ( Supplementary Fig. S12 , Supplementary Tables S17–S20 , available in the online version).

## Discussion

The main finding of this study indicated that the seven bleeding risk scores demonstrated modest predictive accuracy, with the RIETE BRS showing relatively better performance in predicting MB compared with other scores. Patients with low bleeding risk showed improved 14-day composite outcomes with the initial use of DOACs compared with LMWH.

Despite widespread validation in other populations, the Kuijer score showed suboptimal performance in our study. 4 21 22 The BACS score effectively predicted MB in thrombolysis patients, 5 but its performance in anticoagulated patients was limited. The PE-SARD score demonstrated moderate discriminatory ability, similar to the RIETE (AUC = 0.65) and COMMAND-VTE (AUC = 0.64) data, but underperformed in elderly patients (AUC = 0.52), limiting its applicability in specific subgroups. The RIETE BRS in our external validation confirmed an AUC of 0.70, showing modest predictive ability, consistent with previous studies. Lecumberri et al 10 reported an AUC of 0.70 in 82,239 acute VTE patients, and Chopard et al 6 reported 0.69 in the BFC-FRANCE registry. The RIETE BRS demonstrated relatively high sensitivity and NPV, which may help identify low bleeding risk patients, suggesting it could potentially play a role in guiding anticoagulation.

The HAS-BLED and ATRIA scores are primarily used to assess bleeding risk in AF patients and have been widely validated in cardiovascular diseases. 14 23 24 However, their effectiveness in predicting MB risk in PE patients remains controversial. Some studies suggest that the HAS-BLED and ATRIA scores poorly predict MB in PE patients. 25 26 27 The DOAC score developed recently is used to predict bleeding risk in AF patients treated with DOACs, 17 but it has been less evaluated in VTE populations. Although AF and VTE patients differ in clinical complexity and comorbidity burden, they share key bleeding risk factors, such as age, renal function, and history of bleeding. Given the lack of VTE-specific bleeding risk scores recommended in clinical practice guidelines and their current use by physicians for bleeding risk assessment in Pulmonary Thromboembolism (PTE) patients, evaluating the applicability of AF-derived scores in PE patients remains clinically relevant. In this study, we externally tested all three scores derived from the AF population, and the results showed moderate predictive performance, suggesting their potential applicability to PE patients. Since this study focused on validating bleeding risk scores derived from the VTE population and some key variables from AF-derived bleeding scores were missing, further external validation is needed.

Our study indicates that the RIETE BRS showed better reclassification ability than other scores, except HAS-BLED, suggesting its potential role in risk stratification. However, all scores showed limited predictive accuracy in cancer patients, highlighting the need for better tools for these subgroups. This finding is consistent with previous research indicating that traditional scores often lack precision in diverse populations. 28 29 A questionnaire among CURES regional centres ( Supplementary Table S21 , available in the online version) revealed that patients assessed with the RIETE BRS had a lower rate of MB (0.9%) than those evaluated with other scores, indicating its potential to guide anticoagulant treatment in PE better. Future research should focus on enhancing existing scores or developing new ones, particularly for high-risk groups like cancer patients. We emphasize that while metrics like AUC assess predictive performance, clinical decisions should consider individual patient characteristics and other clinical factors. Risk scores should serve as supplementary tools, not the sole basis for decision-making.

We identified anaemia, thrombocytopaenia, and advanced age as independent risk factors for MB in patients with PE, consistent with previous research. 6 30 31 32 Bleeding risk factors varied slightly among patients receiving anticoagulation, with neurological diseases and CTD increasing the risk. Studies have shown that patients with a history of stroke have a significantly higher risk of MB during anticoagulation compared with those without stroke. 33 34 Patients with CTD face elevated bleeding risks, likely due to pathological features, platelet dysfunction, and altered coagulation factors. 35 36 Notably, although we confirmed that active cancer is associated with MB in PE patients, the location, treatment, and stage of cancer also significantly influence bleeding risk. 37 38 This aspect requires further exploration in future studies. Clinicians should consider these factors when developing anticoagulation plans to minimize bleeding and improve outcomes in PE patients. In the CURES cohort, bleeding risk factors differed from those identified in other cohorts, highlighting the need for further research into a PE bleeding risk score tailored to the Chinese population.

Our study supports the importance of optimizing anticoagulant choice to improve patient outcomes. In addition to comorbidities and individual factors, the choice of anticoagulant influences bleeding risk. Patients who experienced MB in our study had much higher risks of adverse outcomes. Research suggests that bleeding risk scores can help guide treatment decisions. 9 39 40 41 Previous studies have shown that DOACs are safe and effective for acute PE and cancer-associated VTE, with better safety profiles than LMWH. 42 43 44 A meta-analysis also found DOACs non-inferior to LMWH for composite outcomes. 45 Furthermore, DOACs are more cost-effective and improve patient adherence, which can help reduce recurrence risk. 46 47 After adjusting for baseline risks, we found that low bleeding risk patients initially treated with DOACs had fewer adverse outcomes at 14 days than those treated with LMWH, and sequential use of DOACs after discharge did not increase the risk of MB or composite outcomes. Therefore, the RIETE BRS might be a useful tool for guiding anticoagulant selection, with DOACs potentially being preferred for low-risk patients due to their safety, cost-effectiveness, and improved adherence, though further validation is required.

The consensus statement on bleeding highlights the interaction between modifiable and non-modifiable bleeding risks, recommending that bleeding risk be assessed at the initiation of anticoagulation and during follow-up. 14 More frequent reassessment is advised for high-risk patients (such as those with a high RIETE BRS), along with closer monitoring and adjustments according to modifiable risk factors. The appropriate use of a validated bleeding risk score is essential. Ethnic differences in bleeding risk, particularly between Asian and Western populations, have been widely acknowledged and may impact both treatment strategies and patient outcomes. 48 The growing body of evidence suggests that bleeding risk scores should be tailored to ethnic populations, particularly in East Asians, where the balance of bleeding and thrombosis risks differs significantly. 19 The East Asian paradox reveals the impact of racial differences on bleeding risks, which is likely a multifactorial phenomenon. Future large-scale prospective studies incorporating genetic, pharmacological, environmental, and clinical factors will help further elucidate the nature of this paradox, providing more substantial personalized evidence for treatment strategies in PE. This also highlights the need for further research and developing bleeding risk scores tailored to different racial groups.

This study has several limitations. Residual confounding may influence the study results, even after adjusting for multiple covariates. Although the cohort is large, its generalizability may be limited, particularly for cancer patients, so bleeding risk scores should be used with caution in these subgroups. Additionally, we could not analyze cancer location or stage due to data limitations, which could significantly affect bleeding risk. Another limitation is that thrombocytopaenia was assessed based on admission platelet counts without further analysis of its severity or underlying causes. Although the RIETE BRS showed better predictive ability than other scores, its overall performance remains modest, classifying patients only into high or intermediate risk, suggesting room for improvement. Moreover, although the bleeding risk scores used at discharge were based on baseline characteristics, the components of the RIETE BRS are unlikely to change significantly in the short term, meaning that the patient's risk is relatively stable. Furthermore, certain factors included in the HAS-BLED and DOAC scores, such as NSAID use and alcohol consumption, were not collected in our dataset. As a result, these factors were assigned a default value of 0 in our analysis, which may have affected the external validity of the models. Finally, our study focused solely on the choice of anticoagulant, as dosing and treatment duration are largely guided by existing guidelines, individual assessments, and ongoing monitoring. Future research should refine risk stratification tools for better personalized anticoagulation and explore their role in determining initial doses and treatment duration.

## Conclusion

This study tested bleeding risk scores derived from VTE or AF populations and showed moderate predictive performance. Among these, the RIETE BRS demonstrated a modest ability to predict MB and might help identify low-risk patients for whom DOACs could be preferable to LMWH for anticoagulation. However, given the modest performance of the score, further studies are required to test these findings and refine the clinical use of bleeding risk scores in the management of acute PE.

## Notes

**What is known about this topic?**

- Bleeding risk assessment is crucial before initiating anticoagulation for PE, with several scores (Kuijer, BACS, PE-SARD, RIETE, HAS-BLED, ATRIA, and DOAC) demonstrating potential effectiveness.
- These scores have not been prospectively validated in large Chinese PE cohorts, and Chinese guidelines still rely on older international protocols.

**What does this paper add?**

- Among the evaluated scores, the RIETE Bleeding Risk Score (BRS) showed modest predictive capability for major bleeding, with an AUC of 0.70, and could potentially be useful in identifying patients at low risk for bleeding.
- For patients classified as low risk by RIETE BRS, initial treatment with DOACs was associated with fewer adverse events within 14 days compared with low-molecular-weight heparin (HR = 0.13, 95% CI, 0.02–0.93).
