# Supplementary material for: Performance of Bleeding Risk Scores for Major Bleeding in Anticoagulated Patients with Pulmonary Embolism: Insights from the CURES Registry-2
Source: Thromb Haemost. 2025 Jul 17;126(5):496–508. doi: 10.1055/a-2642-0241 (PMC13288588; doi:10.1055/a-2642-0241)
Supplement: Supplementary file 1 — Supplementary Material [file 10-1055-a-2642-0241-s24120674.pdf]

Supplementary Method S1 TRIPOD checklist

Section/Topic	Item*		Checklist Item	Page
Title and abstract				
Title	1	V	Identify the study as developing and/or validating a multivariable prediction model, the target population, and the outcome to be predicted.	1
Abstract	2	V	Provide a summary of objectives, study design, setting, participants, sample size, predictors, outcome, statistical analysis, results, and conclusions.	6–7
Introduction				
Background and objectives	3a	V	Explain the medical context (including whether diagnostic or prognostic) and rationale for developing or validating the multivariable prediction model, including references to existing models.	8–9
	3b	V	Specify the objectives, including whether the study describes the development or validation of the model or both.	10
Methods				
Source of data	4a	V	Describe the study design or source of data (e.g., randomized trial, cohort, or registry data), separately for the development and validation data sets, if applicable.	10
	4b	V	Specify the key study dates, including start of accrual, end of accrual, and, if applicable, end of follow-up.	10
Participants	5a	V	Specify key elements of the study setting (e.g., primary care, secondary care, general population) including number and location of centres.	11
	5b	V	Describe eligibility criteria for participants.	10
	5c	V	Give details of treatments received, if relevant.	**►Table 1**
Outcome	6a	V	Clearly define the outcome that is predicted by the prediction model, including how and when assessed.	11, **►Supplementary Method S2**
	6b	V	Report any actions to blind assessment of the outcome to be predicted.	11
Predictors	7a	V	Clearly define all predictors used in developing or validating the multivariable prediction model, including how and when they were measured.	11, **►Supplementary Method S2**, **►Supplementary Table S2**
	7b	V	Report any actions to blind assessment of predictors for the outcome and other predictors.	11
Sample size	8	V	Explain how the study size was arrived at.	Not applicable
Missing data	9	V	Describe how missing data were handled (e.g., complete-case	11, **►Supplementary Method S3**

 © 2025. The Author(s).

Supplementary Method S1 (*Continued*)

Section/Topic	Item*		Checklist Item	Page
			analysis, single imputation, multiple imputation) with details of any imputation method.	
Statistical analysis methods	10a	V	Describe how predictors were handled in the analyses.	11–12
	10b	V	Specify type of model, all model-building procedures (including any predictor selection), and method for internal validation.	Not applicable
	10c	V	For validation, describe how the predictions were calculated.	►**Supplementary Table S2**
	10d	V	Specify all measures used to assess model performance and, if relevant, to compare multiple models.	11–12
	10e	V	Describe any model updating (e.g., recalibration) arising from the validation, if done.	Not applicable
Risk groups	11	V	Provide details on how risk groups were created, if done.	►**Supplementary Table S2**
Development vs. validation	12	V	For validation, identify any differences from the development data in setting, eligibility criteria, outcome, and predictors.	►**Supplementary Method S2**, ►**Supplementary Table S1**
Results				
Participants	13a	V	Describe the flow of participants through the study, including the number of participants with and without the outcome and, if applicable, a summary of the follow-up time. A diagram may be helpful.	13–14, ►**Supplementary Fig. S1**
	13b	V	Describe the characteristics of the participants (basic demographics, clinical features, available predictors), including the number of participants with missing data for predictors and outcome.	13–14, ►**Table 1**
	13c	V	For validation, show a comparison with the development data of the distribution of important variables (demographics, predictors, and outcome).	►**Supplementary Table S1**
Model development	14a	V	Specify the number of participants and outcome events in each analysis.	14, ►**Table 2**, ►**Fig. 1**
	14b	V	If done, report the unadjusted association between each candidate predictor and outcome.	15–16, ►**Supplementary Fig. S7**, ►**Supplementary Fig. S8**, ►**Supplementary Fig. S9**, ►**Supplementary Fig. S10**
Model specification	15a	V	Present the full prediction model to allow predictions for individuals (i.e., all regression coefficients, and model intercept or baseline survival at a given time point).	Not applicable
	15b	V	Explain how to the use the prediction model.	18–19

(*Continued*)

 © 2025. The Author(s).

Supplementary Method S1 (*Continued*)

Section/Topic	Item*		Checklist Item	Page
Model performance	16	V	Report performance measures (with CIs) for the prediction model.	14–16, ►**Table 3**, ►**Table 4**, ►**Supplementary Table S5**, ►**Supplementary Table S6**, ►**Supplementary Table S7**, ►**Supplementary Table S8**, ►**Supplementary Table S9**, ►**Supplementary Table S10**, ►**Supplementary Table S11**, ►**Fig. 2**, ►**Supplementary Fig. S3**, ►**Supplementary Fig. S4**, ►**Supplementary Fig. S5**, ►**Supplementary Fig. S6**
Model updating	17	V	If done, report the results from any model updating (i.e., model specification, model performance).	Not applicable
Discussion				
Limitations	18	V	Discuss any limitations of the study (such as nonrepresentative sample, few events per predictor, missing data).	24–25
Interpretation	19a	V	For validation, discuss the results with reference to performance in the development data, and any other validation data.	19–20
	19b	V	Give an overall interpretation of the results, considering objectives, limitations, results from similar studies, and other relevant evidence.	19–24
Implications	20	V	Discuss the potential clinical use of the model and implications for future research.	22–24
Other information				
Supplementary information	21	V	Provide information about the availability of supplementary resources, such as study protocol, Web calculator, and data sets.	►**Supplementary Material**
Funding	22	V	Give the source of funding and the role of the funders for the present study.	29

*Items relating to a validation of a prediction model are denoted by V.

 © 2025. The Author(s).

Supplementary Method S2 Data collection and variable definitions

Variable	Definition
Cancer	Include both active and inactive types.<br>Active cancer is defined as metastatic cancer or cancer that has received radiotherapy, chemotherapy, immunotherapy, or surgery within the past 6 months.
Immobility	Complete bedrest or inability to walk for 30 minutes per day during ≥3 days.
Recent surgery	Defined as those who had undergone surgery in the 3 months before PE.
Right ventricular dysfunction	Defined by the presence of at least one of the following criteria: echocardiography: an increased end-diastolic RV/LV diameter ≥1.0 in the apical four-chamber view, flattened interventricular septum, TAPSE <16 mm, tricuspid regurgitation pressure gradient >30 mmHg, or abnormal right ventricular wall motion; or CTPA: RV/LV ratio >0.9; or elevated cardiac biomarkers.
Anaemia	For RIETE, ATRIA, and HAS-BLED calculation: haemoglobin level <130 g/L for men and <120 g/L for women.<br>For PE-SARD calculation: haemoglobin level <120 g/L.
Recent bleeding	For RIETE and BACS calculation: recent major bleeding (<90 days prior to PE).
Prior bleeding	For ATRIA calculation: any bleeding (<90 days prior to PE).
Renal dysfunction	For PE-SARD calculation: defined as eGFR <60 mL/min/1.73 m^2.
Severe renal disease	For ATRIA calculation: defined as eGFR <30 mL/min/1.73 m^2.
Abnormal renal function	For HAS-BLED calculation: history of renal impairment diagnosis or serum creatinine ≥200 μmol/L.
Abnormal liver function	For HAS-BLED and DOAC scores calculation: history of hepatic impairment diagnosis, cirrhosis, bilirubin >2× the upper limit of normal, in association with aspartate aminotransferase/alanine aminotransferase >3× the upper limit of normal.
Bleeding history	For DOAC calculation: major or minor bleeding event in the 90 days before PE.
Previous stroke/stroke	Previous history, particularly lacunar.
Uncontrolled hypertension	Uncontrolled, >160 mmHg systolic.
Major bleeding[1]	Fatal bleeding and/or symptomatic bleeding in a critical area or organ (intracranial, intraspinal, intraocular, retroperitoneal, intraarticular or pericardial, or intramuscular with compartment syndrome, and/or bleeding causing a fall in haemoglobin level of 20 g/L [1.24 mmol/L] or more, or leading to transfusion of two or more units of whole blood or red blood cells).
Clinically relevant non-major bleeding[2]	Any sign or symptom of haemorrhage (e.g., more bleeding than would be expected for a clinical circumstance, including bleeding found by imaging alone) that does not fit the criteria for the ISTH definition of MB but does meet at least one of the following criteria: requiring medical intervention by a healthcare professional; leading to hospitalization or increased level of care; prompting a face-to-face (i.e., not just a telephone or electronic communication) evaluation.
Recurrent VTE[3]	Defined as the occurrence of either PE or DVT. For PE: a new intraluminal filling defect in subsegmental or more proximal branches on spiral CT scan; a new intraluminal filling defect, extension of an existing defect, or new sudden vessel cut-off (>2.5 mm) on pulmonary angiogram; a new perfusion defect of at least 75% of a segment with corresponding normal ventilation on ventilation/perfusion lung scintigraphy; or a nondiagnostic lung scan combined with newly diagnosed DVT by ultrasonography or venography. For DVT: in the absence of baseline DVT investigations, a non-compressible venous segment on ultrasonography or an intraluminal filling defect on venography, CT, or MR venography; if baseline DVT investigations are available, recurrence is indicated by abnormal compression ultrasonography where previous results were normal, a substantial increase (≥4 mm) in thrombus size during compression in previously non-compressible veins, or an extension or new intraluminal filling defect or vein non-visualization with a sudden cut-off on venography, CT, or MR venography.
Composite outcome	All-cause death, recurrent VTE, or major bleeding.

Abbreviations: DVT, deep venous thrombosis; eGFR, estimated glomerular filtration rate; ISTH, International Society on Thrombosis and Haemostasis; MB, major bleeding; PE, pulmonary embolism; RV/LV, right ventricle/left ventricle; TAPSE, tricuspid annular plane systolic excursion.
Note: The following variables were collected: sex (male/female), body mass index (BMI), history of VTE (yes/no), cancer (yes/no), recent surgery (yes/no), immobility (yes/no), chronic lung disease (yes/no), chronic obstructive pulmonary disease (COPD, yes/no), congestive heart failure (yes/no), hypertension (yes/no), history of bleeding (yes/no), and right ventricular dysfunction (yes/no). Clinical symptoms at presentation were recorded, including systolic blood pressure, syncope (yes/no), and pulse rate. Laboratory findings included anaemia, platelet count, troponin levels, and renal function markers. All laboratory examinations were performed within 24 hours after admission. Treatment details, such as the use of low-molecular-weight heparin (LMWH), unfractionated heparin, direct oral anticoagulants (DOACs), vitamin K antagonists, thrombolysis, and inferior vena cava filters, were documented. Outcomes were followed up for 90 days, including major bleeding, clinically relevant non-major bleeding, recurrent venous thromboembolism, and overall mortality.

 © 2025. The Author(s).

Supplementary Method S3 The proportion of missing values in CURES-2 data

Variable	Patients, *n* (%)
Body mass index	1,251 (16.4)
Syncope	67 (0.9)
Systolic blood pressure (mmHg)	18 (0.2)
Pulse	129 (1.7)
Platelets	85 (1.1)
Serum creatinine	211 (2.8)
Haemoglobin	136 (1.8)
Aspartate aminotransferase	476 (6.2)
Alanine aminotransferase	323 (4.2)
Total bilirubin	452 (5.9)
International normalized ratio	153 (2.0)

Supplementary Table S1 Baseline characteristics of CURES data compared with those in other studies

	RIETE[4]	Kuijer[5]	PE-SARD[6]	BACS[7]	CURES
	13,057	241	2,754	1,172	7,619
**Clinical characteristics**					
Age, mean ± sd (y)	66 ± 17	63 ± 17	67.3 ± 17.4	63 (47-74)	63 ± 15
Age >75, *n* (%)	NA	NA	1,065 (38.7)	265 (23)	1,737 (22.8)
Sex, male, *n* (%)	6,432 (49)	131 (54)	1,340 (48.7)	570 (49)	3,987 (52.3)
Body mass index (kg/m^2)	NA	NA	27.4 ± 5.9	NA	24.2 ± 3.8
**Risk factors and comorbid diseases, *n* (%)**					
History of VTE	2,088 (16)	NA	NA	NA	1,840 (24.2)
Cancer	2,756 (21)	57 (24)	507 (18.4)[a]	147 (13)[a]	1,098 (14.4)
Recent surgery	1,642 (13)[b]	49 (20)	192 (7.0)	108 (9.2)[c]	906 (11.9)
Immobility	3,252 (25)[d]	NA	NA	254 (22)[e]	621 (8.2)
Chronic lung disease	1,382 (11)	NA	236 (8.6)	NA	1,044 (13.7)
Congestive heart failure	736 (5.6)	NA	NA	58 (4.9)	476 (6.2)
Hypertension	NA	NA	NA	NA	2,749 (36.1)
History of bleeding	NA	NA	42 (1.5)	NA	362 (4.8)
Recent major bleeding	204 (1.6)[f]	NA	NA	14 (1.2)	112 (1.5)
**Clinical symptoms and signs at presentation, *n* (%)**					
Systolic blood pressure (mmHg)	NA	NA	137.7 ± 23.6	NA	127.8 ± 19.6
Syncope	NA	NA	203 (7.4)	527 (36)	585 (7.7)
Pulse ≥110 beats/min	NA	NA	NA	577 (49)	751 (9.9)
Right ventricular dysfunction	NA	NA	911 (33.1)	NA	1,635 (21.5)
Simplified PESI score ≥1	NA	NA	NA	928 (79)	4,820 (63.3)
**2019 ESC-defined risk PE category, *n* (%)**					
High risk	NA	NA	133 (4.8)	NA	450 (5.9)
Intermediate-high risk	NA	NA	584 (21.2)	NA	1,137 (14.9)
Intermediate-low risk	NA	NA	1,594 (57.9)	NA	4,310 (56.6)

 © 2025. The Author(s).

Supplementary Table S1 (*Continued*)

	RIETE[4]	Kuijer[5]	PE-SARD[6]	BACS[7]	CURES
Low risk	NA	NA	443 (16.1)	NA	1,722 (22.6)
**Laboratory findings, *n* (%)**					
Anaemia	4,344 (33)[g]	NA	726 (26.4)[h]	359 (31)[g]	3,025 (39.7)[g]
Platelet count $<100 \times 10^9 \cdot L^{-1}$	305 (2.3)	NA	NA	34 (2.9)	364 (4.8)
Positive troponin	NA	NA	981 (35.6)	NA	1,545 (20.3)
Renal dysfunction	1,891 (14)[i]	NA	825 (29.9)	64 (5.5)[j]	898 (11.8)
**Treatment in the acute phase, *n* (%)**					
LMWH	11,838 (91)	NA	NA	NA	6,052 (79.4)
Unfractioned heparin	1,015 (7.8)	NA	NA	NA	353 (4.6)
Inferior vena cava filter use	297 (2.3)	NA	NA	NA	16 (0.2)
**Outcome, 90 days, *n* (%)**					
Major bleeding	314 (2.4)	9 (3.7)	82 (3.0)[k]	69 (5.9)[l]	107 (1.4)
Clinically relevant non-major bleeding	NA	13 (5.4)	NA	NA	421 (5.5)
Recurrent VTE	302 (2.3)	13 (5.4)	NA	NA	35 (0.5)
Overall death	1,082 (8.3)	NA	NA	94 (8.0)	521 (6.8)

Abbreviations: ESC, European Society of Cardiology; LMWH, low-molecular-weight heparin; NA, not applicable; PE, pulmonary embolism; PESI, Pulmonary Embolism Severity Index; VTE, venous thromboembolism.

[a]Active cancer.

[b]Surgical patients are defined as those who had undergone an operation in the 2 months prior to VTE.

[c]In the previous month.

[d]Immobilized patients are defined as non-surgical patients who had been immobilized (i.e., total bed rest with bathroom privileges) for ≥4 days in the 2-month period prior to VTE diagnosis.

[e]Defined in this analysis as non-surgical patients who had been immobilized (i.e., total bed rest with bathroom privileges) for ≥4 days in the month prior to pulmonary embolism diagnosis.

[f]Major bleeding <15 days prior to VTE.

[g]Anaemia was defined as haemoglobin content <13 g/dL for men and <12 g/dL for women.

[h]Defined by a haemoglobin level <12 g/dL.

[i]Creatinine >1.2 mg/dL.

[j]Creatinine >2 mg/dL.

[k]Median time to event of 2.0 days (Q1–Q3, 1.0–5.0, ranging from 0 to 11 days).

[l]Within the first 30 days of follow-up.

 © 2025. The Author(s).

Supplementary Table S2 Bleeding prediction scores

Score	Kuijer score		RIETE score		PE-SARD score		BACS score		ATRIA score		HAS-BLED score		DOAC score	
Score items (points)	Age >60 years	1	Age >75 years	1	Syncope	1.5	Age >75 years	1	Age ≥75 years	2	Uncontrolled hypertension[a]	1	Age, years	
	Female	1	Creatinine levels >1.2 mg/dL	1.5	Renal dysfunction	1	Recent major bleeding	3	Severe renal disease	3	Abnormal liver/renal function (1 point each)[b]	1/2	65–69<br>70–74<br>75–79<br>≥80	2<br>3<br>4<br>5
	Active cancer	1.5	Anaemia	1.5	Anaemia	2.5	Cancer	1	Anaemia	3	Previous stroke	1		
			Recent bleeding	2			Syncope	1	Prior bleeding	1	Bleeding history or predisposition (anaemia)	1	eGFR (mL/min)	
			Clinically overt PE	1					Hypertension	1	Labile INR (time in therapeutic range <60%)	1	30–60<br><30	1<br>2
			Cancer	1							Age >65 years	1		
											Drugs or alcohol (1 point each)	1/2	Underweight (BMI <18.5)	1
													Stroke/TIA/embolism history	1
													Diabetes	1
													Hypertension	1
													Antiplatelet use	
													Aspirin	2
													Dual-antiplatelet	3
													Nonsteroidal anti-inflammatory drug use	1
													Bleeding history[c]	3
													Liver disease	2
**Original risk categories**														
Low risk	0 point		0 point		0 point		0 point		0–3 points		0–2 points		Very low: 0–3<br>Low: 4–5<br>Moderate: 6–7<br>High: 8–9<br>Very high: 10<br>Total score range: 0–10 (Maximum 10 points)	
Intermediate risk	1–3 points		1–4 points		1–2.5 points		1–3 points		4 points		–			
High risk	>3 points		>4 points		>2.5 points		>3 points		>4 points		≥3 points			

Abbreviations: BMI, body mass index; eGFR, estimated glomerular filtration rate; INR, international normalized ratio; PE, pulmonary embolism; TIA, transient ischaemic attacks.
[a]Uncontrolled, >160 mmHg systolic.
[b]Abnormal kidney function: presence of chronic dialysis, renal transplantation, or serum creatinine ≥200 mmol/L. Abnormal liver function: chronic hepatic disease (e.g., cirrhosis) or biochemical evidence of significant hepatic derangement (e.g., bilirubin >2× upper limit of normal, in association with aspartate aminotransferase/alanine aminotransferase/alkaline phosphatase >3× upper limit normal, and so forth).
[c]History of major or minor bleeding event.

Supplementary Table S3 Collinearity assessment of different bleeding risk scores

Score	Outcome and follow-up	Covariates	VIF
RIETE	14	Thrombolysis	1.142
		2019 ESC risk	1.146
	30	Thrombolysis	1.139
		2019 ESC risk	1.143
	90	Thrombolysis	1.130
		2019 ESC risk	1.135
Kuijer	14	Thrombolysis	1.151
		2019 ESC risk	1.137
	30	Thrombolysis	1.148
		2019 ESC risk	1.135
	90	Thrombolysis	1.141
		2019 ESC risk	1.130
PE-SARD	14	Thrombolysis	1.133
		2019 ESC risk	1.174
	30	Thrombolysis	1.131
		2019 ESC risk	1.172
	90	Thrombolysis	1.125
		2019 ESC risk	1.164
BACS	14	Thrombolysis	1.146
		2019 ESC risk	1.184
	30	Thrombolysis	1.143
		2019 ESC risk	1.179
	90	Thrombolysis	1.136
		2019 ESC risk	1.173

Abbreviation: VIF, variance inflation factors.

 © 2025. The Author(s).

Supplementary Table S4 Predictive value according to bleeding risk scores categories

Score	Outcomes	Unadjusted HR (95% CI)		Adjusted HR (95% CI)[a]	
		High risk vs. low risk	Intermediate risk vs. low risk	High risk vs. low risk	Intermediate risk vs. low risk
RIETE	MB within 14 days	5.79 (3.28–10.23)[b]	NA	5.13 (2.88–9.11)[b]	NA
	MB within 30 days	5.40 (3.18–9.17)[b]	NA	4.86 (2.84–8.31)[b]	NA
	MB within 90 days	4.58 (2.76–7.61)[b]	NA	4.11 (2.47–6.87)[b]	NA
Kuijer	MB within 14 days	5.69 (1.90–17.03)	3.20 (1.16–8.83)	4.66 (1.53–14.20)	2.85 (1.03–7.92)
	MB within 30 days	6.08 (2.05–18.07)	4.06 (1.48–11.15)	5.26 (1.74–15.90)	3.67 (1.33–10.12)
	MB within 90 days	4.61 (1.84–11.54)	3.38 (1.47–7.74)	3.97 (1.56–10.10)	3.07 (1.34–7.07)
PE-SARD	MB within 14 days	4.70 (2.39–9.23)	2.23 (1.32–3.74)	3.57 (1.78–7.12)	1.94 (1.15–3.27)
	MB within 30 days	4.21 (2.23–7.97)	2.33 (1.46–3.72)	3.23 (1.68–6.21)	2.03 (1.26–3.25)
	MB within 90 days	4.01 (2.18–7.37)	2.76 (1.80–4.23)	3.12 (1.67–5.81)	2.44 (1.59–3.75)
BACS	MB within 14 days	24.29 (8.52–69.25)	2.53 (1.56–4.11)	16.36 (5.63–47.51)	2.09 (1.27–3.44)
	MB within 30 days	23.85 (9.36–60.79)	2.27 (1.47–3.52)	16.55 (6.38–42.92)	1.92 (1.23–3.02)
	MB within 90 days	19.47 (7.73–49.06)	2.28 (1.54–3.37)	13.71 (5.36–35.09)	1.95 (1.30–2.92)

Abbreviations: CI, confidence interval; MB, major bleeding.
[a]Adjusted for the 2019 ESC risk stratification and thrombolysis as covariates.
[b]Hazard ratio, high-risk (>4 points) vs. intermediate-risk (≤4 points) groups based on the RIETE bleeding risk score.

Supplementary Table S5 Performance of four bleeding risk scores for major bleeding events at 14, 30, and 90 days

Follow-up	Scores	Sensitivity	Specificity	Predictive value		Likelihood ratio	
		% (95% CI)	% (95% CI)	Positive % (95% CI)	Negative % (95% CI)	Positive (95% CI)	Negative (95% CI)
14 days	RIETE	49.28 (37.02, 61.59)	78.30 (77.34, 79.22)	2.03 (1.73, 2.38)	99.41 (99.21, 99.57)	2.27 (1.62, 3.19)	0.65 (0.46, 0.90)
	Kuijer	86.96 (76.68, 93.86)	29.29 (28.26, 30.33)	1.11 (0.89, 1.38)	99.59 (99.42, 99.72)	1.23 (0.95, 1.59)	0.45 (0.23, 0.86)
	PE-SARD	57.97 (45.47, 69.76)	67.55 (66.48, 68.61)	1.61 (1.33, 1.91)	99.43 (99.24, 99.59)	1.79 (1.31, 2.44)	0.62 (0.43, 0.90)
	BACS	59.42 (46.92, 71.09)	63.98 (62.88, 65.06)	1.49 (1.22, 1.78)	99.42 (99.23, 99.58)	1.65 (1.21, 2.25)	0.63 (0.44, 0.92)
30 days	RIETE	50.00 (39.02, 60.98)	78.91 (77.97, 79.82)	2.64 (2.29, 3.02)	99.28 (99.06, 99.46)	2.37 (1.75, 3.21)	0.63 (0.47, 0.86)
	Kuijer	87.21 (78.27, 93.44)	29.65 (28.61, 30.69)	1.40 (1.14, 1.68)	99.51 (99.33, 99.66)	1.24 (0.99, 1.56)	0.43 (0.24, 0.78)
	PE-SARD	58.14 (47.01, 68.70)	68.12 (67.05, 69.17)	2.04 (1.73, 2.38)	99.30 (99.09, 99.48)	1.82 (1.38, 2.41)	0.61 (0.44, 0.85)
	BACS	59.30 (48.17, 69.78)	64.51 (63.42, 65.60)	1.87 (1.58, 2.21)	99.28 (99.08, 99.47)	1.67 (1.27, 2.20)	0.63 (0.45, 0.88)
90 days	RIETE	49.54 (39.72, 59.37)	80.10 (79.18, 81.00)	3.42 (3.03, 3.86)	99.11 (98.87, 99.31)	2.49 (1.89, 3.27)	0.63 (0.48, 0.82)
	Kuijer	85.94 (77.93, 91.94)	30.28 (29.25, 31.34)	1.73 (1.44, 2.04)	99.34 (99.14, 99.51)	1.23 (1.00, 1.51)	0.46 (0.28, 0.77)
	PE-SARD	61.82 (51.78, 70.92)	68.89 (67.83, 69.94)	2.75 (2.40, 3.15)	99.22 (98.99, 99.40)	1.99 (1.56, 2.54)	0.55 (0.41, 0.75)
	BACS	57.92 (48.01, 67.42)	65.64 (64.56, 66.72)	2.34 (2.02, 2.71)	99.10 (98.86, 99.29)	1.69 (1.31, 2.17)	0.64 (0.48, 0.86)

 © 2025. The Author(s).

Supplementary Table S6 Prediction of major bleeding events at 14, 30, and 90 days using the AF-derived bleeding risk scores

Follow-up	Scores	Sensitivity	Specificity	Predictive value		Likelihood ratio	
		% (95% CI)	% (95% CI)	Positive % (95% CI)	Negative % (95% CI)	Positive (95% CI)	Negative (95% CI)
14 days	HAS-BLED	52.17 (39.80, 64.35)	70.35 (69.30, 71.37)	1.58 (1.32, 1.89)	99.38 (99.18, 99.55)	1.76 (1.27, 2.45)	0.68 (0.48, 0.96)
	ATRIA	53.62 (41.20, 65.72)	79.51 (78.58, 80.42)	2.34 (2.01, 2.70)	99.47 (99.29, 99.62)	2.62 (1.89, 3.63)	0.58 (0.41, 0.83)
	DOAC	62.32 (49.83, 73.71)	61.06 (59.95, 62.16)	1.44 (1.19, 1.74)	99.44 (99.24, 99.59)	1.60 (1.18, 2.16)	0.62 (0.42, 0.91)
30 days	HAS-BLED	55.81 (44.70, 66.52)	70.81 (69.77, 71.83)	2.14 (1.83, 2.49)	99.29 (99.08, 99.47)	1.91 (1.44, 2.55)	0.62 (0.45, 0.86)
	ATRIA	51.16 (40.14, 62.10)	79.89 (78.97, 80.79)	2.82 (2.46, 3.22)	99.31 (99.09, 99.48)	2.54 (1.88, 3.43)	0.61 (0.45, 0.83)
	DOAC	61.63 (50.51, 71.92)	61.40 (60.29, 62.50)	1.79 (1.50, 2.11)	99.29 (99.08, 99.47)	1.60 (1.22, 2.10)	0.62 (0.44, 0.88)
90 days	HAS-BLED	56.067 (46.15, 65.66)	71.34 (70.30, 72.36)	2.71 (2.36, 3.11)	99.13 (98.90, 99.33)	1.96 (1.51, 2.53)	0.62 (0.46, 0.82)
	ATRIA	49.50 (39.72, 59.37)	80.47 (79.56, 81.36)	3.48 (3.08, 3.91)	99.11 (98.87, 99.31)	2.53 (1.93, 3.33)	0.63 (0.48, 0.82)
	DOAC	60.71 (50.84, 70.04)	61.81 (60.70, 62.91)	2.21 (1.90, 2.57)	99.10 (98.87, 99.31)	1.59 (1.24, 2.03)	0.64 (0.47, 0.86)

Supplementary Table S7 Performance of scores for major bleeding at 14, 30, and 90 days in patients receiving anticoagulation

Follow-up	Scores	AUC	Sensitivity	Specificity	Predictive value % (95% CI)		Likelihood ratio (95% CI)	
		(95% CI)	% (95% CI)	% (95% CI)	Positive	Negative	Positive	Negative
14 days	RIETE	0.70 (0.62–0.77)	73.58 (59.67–84.74)	55.89 (54.71–57.08)	1.27 (1.02–1.56)	99.64 (99.47–99.77)	1.67 (1.22–2.29)	0.47 (0.28–0.80)
	Kuijer	0.56 (0.49–0.63)	84.91 (72.41–93.25)	28.95 (27.88–30.03)	0.91 (0.70–1.16)	99.60 (99.42–99.73)	1.19 (0.89–1.60)	0.52 (0.26–1.04)
	PE-SARD	0.63 (0.55–0.70)	58.49 (44.13–71.86)	67.80 (66.69–68.91)	1.38 (1.12–1.69)	99.53 (99.33–99.67)	1.82 (1.27–2.59)	0.61 (0.40–0.93)
	BACS	0.61 (0.53–0.68)	52.83 (38.64–66.70)	64.19 (63.05–65.33)	1.13 (0.89–1.40)	99.44 (99.23–99.60)	1.48 (1.02–2.14)	0.73 (0.50–1.09)
	HAS-BLED	0.68 (0.62–0.74)	54.72 (40.45–68.44)	69.97 (68.88–71.06)	1.39 (1.12–1.69)	99.50 (99.31–99.66)	1.82 (1.26–2.63)	0.65 (0.43–0.97)
	ATRIA	0.69 (0.62–0.76)	56.60 (42.28–70.16)	79.49 (78.51–80.43)	2.08 (1.76–2.45)	99.58 (99.40–99.72)	2.76 (1.92–3.96)	0.55 (0.36–0.82)
	DOAC	0.65 (0.57–0.72)	73.58 (59.67–84.74)	48.11 (46.92–49.30)	1.08 (0.85–1.36)	99.58 (99.40–99.72)	1.42 (1.03–1.94)	0.55 (0.32–0.93)
30 days	RIETE	0.70 (0.64–0.77)	74.63 (62.51–84.47)	56.50 (55.32–57.69)	1.65 (1.36–1.98)	99.56 (99.38–99.71)	1.72 (1.30–2.27)	0.45 (0.28–0.72)
	Kuijer	0.58 (0.52–0.64)	86.57 (76.03–93.67)	29.25 (28.18–30.35)	1.18 (0.94–1.47)	99.55 (99.37–99.70)	1.22 (0.94–1.59)	0.46 (0.24–0.88)
	PE-SARD	0.64 (0.57–0.70)	59.70 (47.00–71.51)	68.39 (67.27–69.48)	1.81 (1.51–2.15)	99.43 (99.21–99.59)	1.89 (1.38–2.58)	0.59 (0.40–0.86)
	BACS	0.61 (0.55–0.68)	53.73 (41.12–66.00)	64.67 (63.52–65.80)	1.46 (1.19–1.77)	99.31 (99.08–99.49)	1.52 (1.09–2.11)	0.72 (0.50–1.02)
	HAS-BLED	0.70 (0.65–0.76)	59.70 (47.00–71.51)	70.39 (69.29–71.46)	1.93 (1.62–2.29)	99.44 (99.23–99.60)	2.02 (1.47–2.76)	0.57 (0.39–0.84)
	ATRIA	0.69 (0.63–0.76)	53.73 (41.12–66.00)	79.80 (78.82–80.74)	2.53 (2.17–2.93)	99.44 (99.23–99.60)	2.66 (1.91–3.70)	0.58 (0.41–0.83)
	DOAC	0.65 (0.59–0.72)	49.25 (36.82–61.76)	72.80 (71.72–73.84)	1.74 (1.44–2.07)	99.32 (99.10–99.50)	1.81 (1.28–2.55)	0.70 (0.50–0.98)
90 days	RIETE	0.71 (0.66–0.77)	75.35 (64.75–84.01)	57.45 (56.28–58.64)	2.15 (1.82–2.52)	99.47 (99.26–99.62)	1.77 (1.38–2.27)	0.43 (0.28–0.66)
	Kuijer	0.58 (0.53–0.64)	84.65 (75.27–91.60)	29.90 (28.82–31.00)	1.48 (1.20–1.79)	99.37 (99.15–99.54)	1.21 (0.96–1.52)	0.51 (0.30–0.89)
	PE-SARD	0.65 (0.60–0.71)	63.67 (52.38–73.71)	69.10 (67.99–70.20)	2.50 (2.14–2.90)	99.35 (99.13–99.53)	2.06 (1.57–2.70)	0.53 (0.37–0.75)
	BACS	0.62 (0.56–0.68)	55.36 (44.11–66.09)	65.80 (64.66–66.92)	1.97 (1.66–2.34)	99.16 (98.92–99.36)	1.62 (1.21–2.16)	0.68 (0.49–0.93)
	HAS-BLED	0.70 (0.65–0.75)	58.78 (47.62–69.39)	70.90 (69.81–71.98)	2.45 (2.10–2.85)	99.28 (99.05–99.46)	2.02 (1.53–2.67)	0.58 (0.42–0.81)
	ATRIA	0.70 (0.65–0.76)	51.73 (40.66–62.74)	80.36 (79.41–81.30)	3.17 (2.78–3.62)	99.26 (99.03–99.45)	2.63 (1.95–3.56)	0.60 (0.44–0.82)
	DOAC	0.64 (0.58–0.70)	60.00 (48.80–70.48)	61.33 (60.16–62.48)	1.89 (1.58–2.24)	99.20 (98.95–99.39)	1.55 (1.18–2.05)	0.65 (0.47–0.91)

 © 2025. The Author(s).

Supplementary Table S8 Comparison of NRI and IDI for various bleeding risk scores with the RIETE score in anticoagulated patients

Score	Follow-up	NRI % (95% CI)			IDI % (95% CI)
		Positive	Negative	Overall	
PE-SARD	14	25.00 (0.00, 50.00)	25.07 (22.69, 27.13)	50.07 (25.29, 75.28)	0.44 (0.21, 0.69)
	30	17.65 (−6.17, 41.67)	26.05 (23.75, 28.39)	43.69 (20.13, 67.41)	0.48 (0.25, 0.75)
	90	20.00 (−1.25, 41.72)	28.60 (26.28, 30.90)	48.60 (27.24, 70.09)	0.48 (0.25, 0.72)
Kuijer	14	32.14 (5.88, 58.31)	25.93 (23.62, 28.28)	58.07 (32.41, 84.81)	0.61 (0.33, 0.90)
	30	26.47 (3.39, 50.34)	31.05 (1.18, 28.66)	57.52 (34.78, 81.46)	0.64 (0.36, 0.94)
	90	31.76 (13.10, 52.88)	28.94 (26.53, 31.29)	60.70 (42.84, 81.55)	0.76 (0.57, 1.16)
BACS	14	21.43 (−4.92, 47.05)	17.72 (15.46, 19.94)	39.15 (12.91, 64.29)	0.05 (−0.32, 0.38)
	30	17.65 (−6.45, 40.83)	18.75 (16.54, 21.02)	36.40 (12.62, 59.30)	−0.05 (−0.47, 0.31)
	90	24.71 (4.00, 46.11)	20.40 (18.09, 22.81)	45.10 (24.26, 66.63)	0.05 (−0.37, 0.42)
HAS-BLED	14	21.47 (−5.36, 43.71)	−19.24 (−21.50, −16.99)	2.23 (−24.16, 24.74)	0.13 (−0.27, 0.47)
	30	14.58 (−7.64, 38.61)	−34.25 (−36.31, −32.01)	−19.67 (−42.51, 5.05)	−0.03 (−0.62, 0.35)
	90	22.75 (2.65, 43.52)	−31.20 (−36.28, −31.92)	−11.45 (−31.77, 9.59)	0.10 (−0.31, 0.51)
ATRIA	14	−10.48 (−36.31, 16.12)	58.26 (56.38, 60.23)	47.78 (22.35, 74.08)	0.28 (0.05, 0.51)
	30	−11.46 (−35.38, 12.06)	58.33 (56.25, 60.54)	46.87 (23.28, 70.05)	0.33 (0.12, 0.48)
	90	−17.31 (−37.37, 3.30)	58.38 (56.47, 60.41)	41.08 (20.66, 61.66)	0.29 (0.08, 0.53)
DOAC	14	21.64 (−2.91, 46.23)	24.26 (22.06, 26.54)	45.89 (20.63, 70.48)	0.39 (0.16, 0.63)
	30	14.97 (−9.33, 37.64)	24.36 (22.04, 26.74)	39.33 (14.86, 63.33)	0.37 (0.12, 0.61)
	90	18.35 (−3.32, 39.71)	24.42 (22.11, 26.83)	42.77 (21.29, 64.44)	0.49 (0.25, 0.79)

Abbreviations: IDI, integrated discrimination improvement; NRI, net reclassification improvement.

Supplementary Table S9 Performance of bleeding risk scores for major bleeding at 14, 30, and 90 days in non-cancer patients receiving anticoagulation

Follow-up	Scores	AUC	Sensitivity	Specificity	Predictive value % (95% CI)		Likelihood ratio (95% CI)	
		(95% CI)	% (95% CI)	% (95% CI)	Positive	Negative	Positive	Negative
14 days	RIETE	0.70 (0.61–0.79)	72.50 (56.11–85.40)	59.53 (58.26–60.79)	1.21 (0.94–1.51)	99.69 (99.50–99.81)	1.79 (1.24–2.58)	0.46 (0.26–0.83)
	Kuijer	0.54 (0.47–0.62)	80.00 (64.35–90.95)	33.78 (32.57–35.01)	0.82 (0.60–1.07)	99.60 (99.40–99.74)	1.21 (0.85–1.71)	0.59 (0.30–1.19)
	PE-SARD	0.64 (0.56–0.73)	57.50 (40.89–72.96)	70.78 (69.60–71.94)	1.32 (1.04–1.64)	99.59 (99.40–99.74)	1.97 (1.30–2.97)	0.60 (0.37–0.97)
	BACS	0.59 (0.51–0.67)	42.50 (27.04–59.11)	72.39 (71.22–73.52)	1.04 (0.79–1.32)	99.46 (99.24–99.63)	1.54 (0.95–2.48)	0.79 (0.53–1.20)
	HAS-BLED	0.70 (0.63–0.77)	57.50 (40.89–72.96)	70.56 (69.38–71.72)	1.31 (1.04–1.64)	99.59 (99.40–99.74)	1.95 (1.29–2.95)	0.60 (0.37–0.97)
	ATRIA	0.71 (0.62–0.79)	57.50 (40.89–72.96)	80.60 (79.57–81.61)	1.98 (1.64–2.37)	99.64 (99.46–99.78)	2.96 (1.96–4.48)	0.53 (0.33–0.85)
	DOAC	0.67 (0.58–0.76)	67.50 (50.87–81.43)	59.92 (58.65–61.17)	1.13 (0.88–1.44)	99.63 (99.44–99.77)	1.68 (1.15–2.46)	0.54 (0.31–0.93)
30 days	RIETE	0.70 (0.62–0.78)	72.55 (58.26–84.11)	59.93 (58.66–61.19)	1.55 (1.26–1.90)	99.60 (99.42–99.75)	1.81 (1.31–2.51)	0.46 (0.27–0.77)
	Kuijer	0.57 (0.51–0.63)	82.35 (69.13–91.60)	33.99 (32.77–35.21)	1.07 (0.82–1.36)	99.55 (99.34–99.70)	1.25 (0.92–1.69)	0.52 (0.27–1.00)
	PE-SARD	0.64 (0.57–0.72)	56.86 (42.25–70.65)	71.08 (69.91–72.24)	1.68 (1.36–2.03)	99.47 (99.26–99.64)	1.97 (1.36–2.84)	0.61 (0.40–0.92)
	BACS	0.60 (0.52–0.67)	43.14 (29.35–57.75)	72.55 (71.39–73.69)	1.35 (1.07–1.68)	99.32 (99.08–99.52)	1.57 (1.03–2.39)	0.78 (0.54–1.13)
	HAS-BLED	0.72 (0.66–0.78)	62.75 (48.08–75.87)	70.92 (69.73–72.07)	1.84 (1.52–2.22)	99.55 (99.34–99.70)	2.16 (1.52–3.06)	0.53 (0.33–0.82)
	ATRIA	0.70 (0.63–0.77)	52.94 (38.46–67.07)	80.85 (79.83–81.86)	2.35 (1.98–2.77)	99.50 (99.28–99.66)	2.77 (1.89–4.05)	0.58 (0.39–0.87)
	DOAC	0.67 (0.60–0.75)	54.90 (40.34–68.87)	72.08 (70.91–73.22)	1.68 (1.36–2.03)	99.46 (99.24–99.63)	1.97 (1.35–2.86)	0.63 (0.42–0.94)
90 days	RIETE	0.71 (0.64–0.78)	73.93 (61.46–83.97)	60.31 (59.04–61.56)	2.03 (1.69–2.42)	99.52 (99.32–99.69)	1.86 (1.40–2.48)	0.43 (0.27–0.70)
	Kuijer	0.57 (0.51–0.63)	79.92 (68.23–88.90)	34.19 (32.98–35.43)	1.33 (1.06–1.66)	99.35 (99.12–99.54)	1.21 (0.92–1.60)	0.59 (0.34–1.01)
	PE-SARD	0.66 (0.60–0.72)	61.71 (48.64–73.35)	71.40 (70.21–72.55)	2.34 (1.96–2.75)	99.41 (99.18–99.59)	2.16 (1.58–2.95)	0.54 (0.36–0.80)
	BACS	0.60 (0.54–0.67)	44.68 (32.27–57.47)	72.74 (71.58–73.88)	1.79 (1.47–2.16)	99.16 (98.89–99.37)	1.64 (1.14–2.37)	0.76 (0.55–1.06)
	HAS-BLED	0.72 (0.67–0.78)	64.65 (51.77–76.08)	71.32 (70.15–72.48)	2.44 (2.07–2.88)	99.45 (99.24–99.63)	2.25 (1.66–3.06)	0.50 (0.33–0.75)
	ATRIA	0.71 (0.65–0.78)	52.32 (39.54–64.85)	81.21 (80.18–82.20)	3.00 (2.59–3.48)	99.35 (99.12–99.54)	2.79 (1.98–3.92)	0.59 (0.41–0.84)
	DOAC	0.67 (0.60–0.73)	66.18 (53.35–77.43)	60.64 (59.37–61.89)	1.83 (1.50–2.20)	99.38 (99.16–99.57)	1.68 (1.24–2.27)	0.56 (0.37–0.85)

 © 2025. The Author(s).

Supplementary Table S10 Performance of bleeding risk scores for major bleeding at 14, 30, and 90 days in cancer patients receiving anticoagulation

Follow-up	Scores	Cancer	Active cancer
		AUC (95% CI)	AUC (95% CI)
14 days	RIETE	0.58 (0.43–0.73)	0.53 (0.35–0.71)
	Kuijer	0.47 (0.30–0.65)	0.44 (0.23–0.66)
	PE-SARD	0.53 (0.40–0.66)	0.46 (0.31–0.62)
	BACS	0.54 (0.39–0.69)	0.57 (0.42–0.72)
	HAS-BLED	0.61 (0.46–0.75)	0.58 (0.41–0.74)
	ATRIA	0.60 (0.45–0.76)	0.58 (0.39–0.77)
	DOAC	0.59 (0.45–0.73)	0.60 (0.45–0.76)
30 days	RIETE	0.60 (0.48–0.72)	0.56 (0.42–0.70)
	Kuijer	0.44 (0.29–0.60)	0.42 (0.24–0.60)
	PE-SARD	0.57 (0.46–0.69)	0.53 (0.40–0.66)
	BACS	0.54 (0.42–0.66)	0.53 (0.41–0.65)
	HAS-BLED	0.62 (0.50–0.75)	0.61 (0.47–0.74)
	ATRIA	0.64 (0.50–0.77)	0.63 (0.47–0.78)
	DOAC	0.59 (0.45–0.73)	0.60 (0.45–0.76)
90 days	RIETE	0.59 (0.49–0.70)	0.56 (0.45–0.68)
	Kuijer	0.44 (0.30–0.59)	0.43 (0.27–0.59)
	PE-SARD	0.59 (0.48–0.69)	0.55 (0.44–0.67)
	BACS	0.55 (0.45–0.64)	0.51 (0.42–0.61)
	HAS-BLED	0.59 (0.49–0.70)	0.58 (0.47–0.69)
	ATRIA	0.63 (0.52–0.74)	0.62 (0.49–0.75)
	DOAC	0.55 (0.42–0.68)	0.56 (0.42–0.70)

Supplementary Table S11 Sensitivity analysis investigating the discriminative performance of bleeding risk scores derived from the VTE population

Follow-up	Scores	All patients (7,619)		Patients with anticoagulation (6,921)	
		Remove patients with missing values	Remove patients lost to follow-up within 90 days	Remove patients with missing values	Remove patients lost to follow-up within 90 days
14 days	RIETE	0.69 (0.62–0.75)	0.69 (0.62–0.75)	0.70 (0.62–0.77)	0.70 (0.62–0.77)
	Kuijer	0.60 (0.54–0.67)	0.60 (0.54–0.66)	0.56 (0.49–0.63)	0.56 (0.49–0.63)
	PE-SARD	0.64 (0.57–0.71)	0.64 (0.58–0.71)	0.62 (0.55–0.70)	0.63 (0.55–0.70)
	BACS	0.64 (0.57–0.70)	0.63 (0.57–0.70)	0.61 (0.53–0.69)	0.61 (0.53–0.68)
30 days	RIETE	0.69 (0.63–0.75)	0.69 (0.63–0.75)	0.70 (0.64–0.77)	0.70 (0.64–0.77)
	Kuijer	0.60 (0.55–0.66)	0.60 (0.55–0.66)	0.58 (0.52–0.64)	0.58 (0.52–0.64)
	PE-SARD	0.65 (0.59–0.70)	0.65 (0.59–0.70)	0.63 (0.57–0.70)	0.64 (0.57–0.70)
	BACS	0.64 (0.58–0.69)	0.63 (0.58–0.69)	0.61 (0.55–0.68)	0.61 (0.54–0.68)
90 days	RIETE	0.70 (0.65–0.75)	0.70 (0.65–0.75)	0.71 (0.65–0.77)	0.71 (0.65–0.77)
	Kuijer	0.61 (0.56–0.66)	0.60 (0.56–0.65)	0.59 (0.53–0.64)	0.58 (0.53–0.64)
	PE-SARD	0.66 (0.61–0.71)	0.66 (0.61–0.71)	0.65 (0.59–0.71)	0.65 (0.60–0.71)
	BACS	0.63 (0.58–0.69)	0.63 (0.58–0.68)	0.63 (0.57–0.68)	0.62 (0.56–0.68)

 © 2025. The Author(s).

Supplementary Table S12 Impact of major bleeding on 90-day outcomes in patients with pulmonary embolism

Outcome	HR (95% CI)	sHR (95% CI)[a]
Death, 90 days	6.07 (4.11–8.95)	4.12 (2.78–6.11)
Composite events, 90 days[b]	6.01 (4.07–8.86)	4.34 (2.93–6.42)

[a]Hazard ratio, adjusted for age, sex, pulmonary embolism (PE) severity, and active cancer as covariates.
[b]Includes venous thromboembolism (VTE) recurrence and all-cause mortality.

Supplementary Table S13 Standardized mean difference before and after propensity score matching in patients receiving LMWH or DOACs at admission

	High risk[a]		Low risk[a]	
Variables	Before PSM	After PSM	Before PSM	After PSM
Prior major bleeding ’0	0.1132	0.0938	NA	NA
Prior major bleeding ’1	−0.1132	−0.0938	NA	NA
Elder >65 ’0	−0.3170	−0.0513	0.0032	−0.0112
Elder >65 ’1	0.3170	0.0513	−0.0032	0.0112
Active malignancy ’0	0.3191	−0.0388	0.1568	−0.0240
Active malignancy ’1	−0.3191	0.0388	−0.1568	0.0240
Gastrointestinal diseases ’0	0.0314	0.0316	−0.0574	−0.0157
Gastrointestinal diseases ’1	−0.0314	−0.0316	0.0574	0.0157
Anaemia ’0	0.0584	−0.0854	0.0757	0.0035
Anaemia ’1	−0.0584	0.0854	−0.0757	−0.0035
CR >1.2 mg/dL ’0	−0.2366	−0.0306	0.0583	−0.0236
CR >1.2 mg/dL ’1	0.2366	0.0306	−0.0583	0.0236
PLT $<100 \times 10^9$/L ’0	0.0623	−0.0573	0.1294	−0.0194
PLT $<100 \times 10^9$/L ’1	−0.0623	0.0573	−0.1294	0.0194
Pulse >100 beats/min ’0	0.2713	−0.0737	0.1883	−0.0123
Pulse >100 beats/min ’1	−0.2713	0.0737	−0.1883	0.0123
Connective tissue disease ’0	0.0293	0	−0.0008	−0.0310
Connective tissue disease ’1	−0.0293	0	0.0008	0.0310
Neurological disease ’0	0.0778	−0.0184	0.0049	0.0096
Neurological disease ’1	−0.0778	0.0184	−0.0049	−0.0096
2019 ESC-defined risk categories				
High risk	−0.0837	0.0669	0.0277	−0.0016
Intermediate-high risk	−0.0792	−0.0194	−0.2049	−0.0138
Intermediate-low risk	−0.0100	−0.0140	−0.1202	−0.0065
Low risk	0.1549	−0.0067	0.2323	0.0155

Abbreviations: CR, creatinine; DOACs, direct oral anticoagulants; LMWH, low-molecular-weight heparin; PLT, platelet; PSM, propensity score matching.
Note: NA: In patients stratified as low bleeding risk according to the re-classified RIETE score, no individuals experienced major bleeding within the past 3 months. ‘0’ indicates absence of the condition; ‘1’ indicates presence of the condition.
[a]After stratifying by the RIETE score into low and high bleeding risk groups, PSM was conducted between the DOACs and LMWH groups.

Supplementary Table S14 Comparison of LMWH and DOACs in low bleeding risk patients based on RIETE bleeding risk score

Subjects	Before PSM ($N = 4{,}930$)			After PSM ($N = 2{,}521$)		
	LMWH	DOACs	*P*-value	LMWH	DOACs	*P*-value
	$N = 4{,}421$ *n* (%)	$N = 509$ *n* (%)		$N = 2{,}012$ *n* (%)	$N = 509$ *n* (%)	
Age >65 years	2083 (47.1)	239 (47.0)	0.982	925 (46.0)	239 (47.0)	0.729
Active cancer	235 (5.3)	14 (2.8)	0.017[a]	48 (2.4)	14 (2.8)	0.753
Recent major bleeding	0	0	NA	0	0	NA
Gastrointestinal diseases	232 (5.2)	34 (6.7)	0.211	115 (5.7)	34 (6.7)	0.472
ESC-defined risk categories			<0.001[a]			0.936
High risk	158 (3.6)	21 (4.1)		76 (3.8)	21 (4.1)	
Intermediate-high risk	648 (14.7)	45 (8.8)		188 (9.3)	45 (8.8)	
Intermediate-low risk	2437 (55.1)	250 (49.1)		1006 (50.0)	250 (49.1)	
Low risk	1178 (26.6)	193 (37.9)		742 (36.9)	193 (37.9)	
Anaemia	1157 (26.2)	117 (23.0)	0.133	468 (23.3)	117 (23.0)	0.942
Platelet count $<100 \times 10^9$/L	191 (4.3)	12 (2.4)	0.046[a]	42 (2.1)	12 (2.4)	0.838
Serum creatinine >1.2 mg/dL	133 (3.0)	1 (2.2)	0.349	37 (1.8)	1 (2.2)	0.769
Pulse >100 beats/min	779 (17.6)	59 (11.6)	0.001[a]	227 (11.3)	59 (11.6)	0.906
Connective tissue disease	147 (3.3)	17 (3.3)	0.999	52 (2.6)	17 (3.3)	0.435
Neurological disease	467 (10.6)	53 (10.4)	0.977	213 (10.6)	53 (10.4)	0.973
Outcome						
Major bleeding (14 days)	25 (0.6)	0 (0.0)	0.170	10 (0.5)	0 (0.0)	0.231
Major bleeding (30 days)	32 (0.7)	0 (0.0)	0.102	10 (0.5)	0 (0.0)	0.231
Major bleeding (90 days)	38 (0.9)	0 (0.0)	0.067	12 (0.6)	0 (0.0)	0.166
Composite outcome (14 days)	86 (1.9)	1 (0.2)	0.008[a]	31 (1.5)	1 (0.2)	0.028[a]
Composite outcome (30 days)	146 (3.3)	8 (1.6)	0.046[a]	47 (2.3)	8 (1.6)	0.376
Composite outcome (90 days)	237 (5.4)	14 (2.8)	0.015[a]	85 (4.2)	14 (2.8)	0.161

Abbreviations: CR, creatinine; DOACs, direct oral anticoagulants; LMWH, low-molecular-weight heparin; PLT, platelet count; PSM, propensity score matching.
Notes: NA: In patients stratified as low bleeding risk according to the re-classified RIETE score, no individuals experienced major bleeding within the past 3 months.
[a]Statistically significant.

 © 2025. The Author(s).

Supplementary Table S15 Comparison of clinical outcomes between initial therapy with DOACs and LMWH in propensity score-matched cohorts at admission

Outcome	High bleeding risk group	Low bleeding risk group
	HR (95% CI)[a]	HR (95% CI)[a]
MB within 14 days	0.70 (0.08, 6.00)	NA
Composite events within 14 days	0.54 (0.12, 2.40)	0.13 (0.02, 0.93)
MB within 30 days	0.58 (0.07, 4.86)	NA
Composite events within 30 days	0.77 (0.32, 1.85)	0.67 (0.32, 1.41)
MB within 90 days	1.01 (0.21, 4.85)	NA
Composite events within 90 days	1.03 (0.58, 1.82)	0.65 (0.37, 1.13)

Abbreviations: CI, confidence interval; DOACs, direct oral anticoagulants; LMWH, low-molecular-weight heparin; MB, major bleeding.
Note: NA: Calculation could not be performed due to the small sample size of major bleeding cases.
[a]Hazard ratio, DOACs vs. LMWH (reference group).

Supplementary Table S16 Comparison of LMWH and DOACs before and after propensity score matching in high bleeding risk patients based on RIETE bleeding risk score at admission

Subjects	Before PSM ($N = 1,415$)			After PSM ($N = 531$)		
	LMWH	DOACs	*P*-value	LMWH	DOACs	*P*-value
	$N = 1,293$ *n* (%)	$N = 122$ *n* (%)		$N = 413$ *n* (%)	$N = 118$ *n* (%)	
Age >65 years	943 (72.9)	103 (84.4)	0.008[a]	337 (81.6)	99 (83.9)	0.661
Active cancer	483 (37.4)	29 (23.8)	0.004[a]	100 (24.2)	29 (24.6)	0.999
Recent major bleeding	82 (6.3)	5 (4.1)	0.430	11 (2.7)	4 (3.4)	0.916
Gastrointestinal diseases	106 (8.2)	9 (7.4)	0.886	31 (7.5)	8 (6.8)	0.947
ESC-defined risk categories			0.163			0.362
High risk	87 (6.7)	6 (4.9)		15 (3.6)	5 (4.2)	
Intermediate-high risk	181 (14.0)	14 (11.5)		43 (10.4)	13 (11.0)	
Intermediate-low risk	949 (73.4)	89 (73.0)		334 (80.9)	89 (75.4)	
Low risk	76 (5.9)	13 (10.7)		21 (5.1)	11 (9.3)	
Anaemia	1,129 (87.3)	104 (85.2)	0.609	355 (86.0)	101 (85.6)	0.999
Platelet count $<100 \times 10^9$	81 (6.3)	6 (4.9)	0.693	15 (3.6)	5 (4.2)	0.976
Serum creatinine >1.2 mg/dL	290 (22.4)	41 (33.6)	0.007[a]	107 (25.9)	39 (33.1)	0.157
Pulse >100 beats/min	246 (19.0)	13 (10.7)	0.031[a]	30 (7.3)	13 (11.0)	0.260
Connective tissue disease	26 (2.0)	2 (1.6)	0.999	4 (1.0)	2 (1.7)	0.869
Neurological disease	215 (16.6)	17 (13.9)	0.522	44 (10.7)	15 (12.7)	0.645
Outcome						
Major bleeding (14 days)	25 (1.9)	1 (0.8)	0.601	5 (1.2)	1 (0.8)	0.999
Major bleeding (30 days)	29 (2.2)	1 (0.8)	0.475	6 (1.5)	1 (0.8)	0.959
Major bleeding (90 days)	35 (2.7)	2 (1.6)	0.682	7 (1.7)	2 (1.7)	0.999
Composite outcome (14 days)	54 (4.2)	2 (1.6)	0.258	13 (3.1)	2 (1.7)	0.600
Composite outcome (30 days)	120 (9.3)	6 (4.9)	0.147	27 (6.5)	6 (5.1)	0.729
Composite outcome (90 days)	230 (17.8)	15 (12.3)	0.159	52 (12.6)	15 (12.7)	0.999

Abbreviations: CR, creatinine; DOACs, direct oral anticoagulants; LMWH, low-molecular-weight heparin; PLT, platelet count; PSM, propensity score matching.
[a]Statistically significant.

 © 2025. The Author(s).

Supplementary Table S17 Standardized mean difference before and after propensity score matching in patients receiving LMWH or DOACs at discharge

	High risk[a]		Low risk[a]	
Variables	Before PSM	After PSM	Before PSM	After PSM
Prior major bleeding '0	0.0003	−0.0291	NA	NA
Prior major bleeding '1	−0.0003	0.0291	NA	NA
Elder >65 '0	0.0307	0.0000	−0.0315	−0.0022
Elder >65 '1	−0.0307	0.0000	0.0315	0.0022
Active malignancy '0	−0.2302	−0.0070	−0.0632	0.0000
Active malignancy '1	0.2302	0.0070	0.0632	0.0000
Gastrointestinal diseases '0	−0.0143	0.0117	−0.0592	−0.0071
Gastrointestinal diseases '1	0.0143	−0.0117	0.0592	0.0071
Anaemia '0	−0.1137	0.0097	−0.0368	0.0038
Anaemia '1	0.1137	−0.0097	0.0368	−0.0038
CR >1.2 mg/dL '0	0.0040	0.0143	0.0532	0.0000
CR >1.2 mg/dL '1	−0.0040	−0.0143	−0.0532	0.0000
PLT <100 × 10^9/L '0	−0.0940	0.0263	0.0109	0.0029
PLT <100 × 10^9/L '1	0.0940	−0.0263	−0.0109	−0.0029
Pulse >100 beats/min '0	0.0393	0.0000	0.0543	0.0061
Pulse >100 beats/min '1	−0.0393	0.0000	−0.0543	−0.0061
Connective tissue disease '0	0.0615	0.0000	0.0291	−0.0033
Connective tissue disease '1	−0.0615	0.0000	−0.0291	0.0033
Neurological disease '0	−0.0554	0.0000	−0.0695	0.0070
Neurological disease '1	0.0554	0.0000	0.0695	−0.0070
2019 ESC-defined risk categories				
High risk	0.0296	0.0295	0.0598	0.0000
Intermediate-high risk	−0.1589	−0.0092	−0.0909	0.0016
Intermediate-low risk	0.1266	0.0000	−0.0504	−0.0022
Low risk	−0.0266	−0.0125	0.0960	0.0012

Abbreviations: CR, creatinine; DOACs, direct oral anticoagulants; LMWH, low-molecular-weight heparin; PLT, platelet; PSM, propensity score matching.
Note: NA: In patients stratified as low bleeding risk according to the re-classified RIETE score, no individuals experienced major bleeding within the past 3 months.
[a]After stratifying by the RIETE score into low and high bleeding risk groups, PSM was conducted between the DOACs and LMWH groups.

 © 2025. The Author(s).

Supplementary Table S18 Comparison of LMWH and DOACs before and after propensity score matching in high bleeding risk patients based on RIETE bleeding risk score at discharge

Subjects	Before PSM (*N* = 1,030)			After PSM (*N* = 628)		
	LMWH	DOACs	*P*-value	LMWH	DOACs	*P*-value
	N = 376 *n* (%)	*N* = 654 *n* (%)		*N* = 314 *n* (%)	*N* = 314 *n* (%)	
Age >65 years	298 (79.3)	510 (78.0)	0.689	258 (82.2)	258 (82.2)	0.999
Active cancer	72 (19.1)	194 (29.7)	<0.001[a]	64 (20.4)	65 (20.7)	0.999
Recent major bleeding	19 (5.1)	33 (5.0)	0.999	4 (1.3)	6 (1.9)	0.750
Gastrointestinal diseases	29 (7.7)	53 (8.1)	0.917	17 (5.4)	16 (5.1)	0.999
ESC-defined risk categories			0.116			0.968
High risk	16 (4.3)	32 (4.9)		8 (2.5)	10 (3.2)	
Intermediate-high risk	73 (19.4)	91 (13.9)		55 (17.5)	54 (17.2)	
Intermediate-low risk	258 (68.6)	485 (74.2)		232 (73.9)	232 (73.9)	
Low risk	29 (7.7)	46 (7.0)		19 (6.1)	18 (5.7)	
Anaemia	316 (84.0)	574 (87.8)	0.113	279 (88.9)	278 (88.5)	0.999
Platelet count $<100 \times 10^9$	15 (4.0)	41 (6.3)	0.158	10 (3.2)	8 (2.5)	0.811
Serum creatinine >1.2 mg/dL	103 (27.4)	178 (27.2)	0.999	80 (25.5)	78 (24.8)	0.927
Pulse >100 beats/min	64 (17.0)	102 (15.6)	0.609	38 (12.1)	38 (12.1)	0.999
Connective tissue disease	10 (2.7)	12 (1.8)	0.511	3 (1.0)	3 (1.0)	0.999
Neurological disease	50 (13.3)	100 (15.3)	0.435	31 (9.9)	31 (9.9)	0.999
Outcome						
Major bleeding (14 days)	1 (0.3)	11 (1.7)	0.082	0 (0.0)	3 (1.0)	0.247
Major bleeding (30 days)	3 (0.8)	12 (1.8)	0.286	2 (0.6)	3 (1.0)	0.999
Major bleeding (90 days)	5 (1.3)	16 (2.4)	0.321	4 (1.3)	5 (1.6)	0.999
Composite outcome (14 days)	4 (1.1)	11 (1.7)	0.598	3 (1.0)	3 (1.0)	0.999
Composite outcome (30 days)	16 (4.3)	30 (4.6)	0.927	15 (4.8)	14 (4.5)	0.999
Composite outcome (90 days)	33 (8.8)	71 (10.9)	0.337	30 (9.6)	34 (10.8)	0.692

Abbreviations: CR, creatinine; DOACs, direct oral anticoagulants; LMWH, low-molecular-weight heparin; PLT, platelet count; PSM, propensity score matching.
[a]Statistically significant.

 © 2025. The Author(s).

Supplementary Table S19 Comparison of LMWH and DOACs before and after propensity score matching in low bleeding risk patients based on RIETE bleeding risk score at discharge

Subjects	Before PSM ($N = 4,600$)			After PSM ($N = 3,644$)		
	LMWH	DOACs	*P*-value	LMWH	DOACs	*P*-value
	$N = 1,933$ *n* (%)	$N = 2,667$ *n* (%)		$N = 1,822$ *n* (%)	$N = 1,822$ *n* (%)	
Age >65 years	893 (46.2)	1,274 (47.8)	0.306	857 (47.0)	859 (47.1)	0.974
Active cancer	45 (2.3)	93 (3.5)	0.029[a]	43 (2.4)	43 (2.4)	0.999
Recent major bleeding	0	0	NA	0	0	NA
Gastrointestinal diseases	83 (4.3)	151 (5.7)	0.044[a]	62 (3.4)	65 (3.6)	0.857
ESC-defined risk categories			<0.001[a]			0.999
High risk	53 (2.7)	104 (3.9)		43 (2.4)	43 (2.4)	
Intermediate-high risk	313 (16.2)	350 (13.1)		268 (14.7)	269 (14.8)	
Intermediate-low risk	1,059 (54.8)	1,394 (52.3)		1,016 (55.8)	1,014 (55.7)	
Low risk	508 (26.3)	819 (30.7)		495 (27.2)	496 (27.2)	
Anaemia	459 (23.7)	676 (25.3)	0.227	429 (23.5)	426 (23.4)	0.938
Platelet count $<100 \times 10^9$	75 (3.9)	98 (3.7)	0.777	36 (2.0)	35 (1.9)	0.999
Serum creatinine >1.2 mg/dL	68 (3.5)	71 (2.7)	0.113	45 (2.5)	45 (2.5)	0.999
Pulse >100 beats/min	335 (17.3)	410 (15.4)	0.082	283 (15.5)	279 (15.3)	0.891
Connective tissue disease	66 (3.4)	78 (2.9)	0.392	34 (1.9)	35 (1.9)	0.999
Neurological disease	173 (8.9)	297 (11.1)	0.018[a]	150 (8.2)	146 (8.0)	0.856
Outcome						
Major bleeding (14 days)	2 (0.1)	8 (0.3)	0.275	2 (0.1)	6 (0.3)	0.288
Major bleeding (30 days)	4 (0.2)	10 (0.4)	0.453	4 (0.2)	7 (0.4)	0.546
Major bleeding (90 days)	8 (0.4)	13 (0.5)	0.886	8 (0.4)	10 (0.5)	0.813
Composite outcome (14 days)	9 (0.5)	12 (0.4)	0.999	9 (0.5)	8 (0.4)	0.999
Composite outcome (30 days)	20 (1.0)	38 (1.4)	0.300	18 (1.0)	24 (1.3)	0.438
Composite outcome (90 days)	48 (2.5)	76 (2.8)	0.506	38 (2.1)	49 (2.7)	0.278

Abbreviations: CR, creatinine; DOACs, direct oral anticoagulants; LMWH, low-molecular-weight heparin; PLT, platelet count; PSM, propensity score matching.
[a]Statistically significant.

 © 2025. The Author(s).

Supplementary Table S20 Comparison of clinical outcomes between DOACs and LMWH in propensity score-matched cohorts at discharge

Outcome	High bleeding risk group	Low bleeding risk group
	HR (95% CI)[a]	HR (95% CI)[a]
MB within 14 days	NA	3.00 (0.33, 14.90)
Composite events within 14 days	1.00 (0.20, 4.95)	1.12 (0.34, 2.30)
MB within 30 days	1.51 (0.25, 8.99)	1.75 (0.51, 5.98)
Composite events within 30 days	0.94 (0.45, 1.94)	1.33 (0.73, 2.46)
MB within 90 days	1.25 (0.34, 4.64)	1.25 (0.49, 3.16)
Composite events within 90 days	1.13 (0.69, 1.84)	1.29 (0.84, 1.96)

Abbreviations: CI, confidence interval; DOACs, direct oral anticoagulants; LMWH, low-molecular-weight heparin; MB, major bleeding.
Notes: NA: Calculation could not be performed due to the small sample size of major bleeding cases.
[a]Hazard ratio, DOACs vs. LMWH (reference group).

Supplementary Table S21 Comparison of major bleeding rates by bleeding risk scores in patients with PE based on questionnaire findings

Scores	Total patients	Major bleeding (%)[a]	*P*-value
2012 ACCP	1,535	45 (2.9)	Reference
RIETE	416	4 (0.9)	0.036
HAS-BLED	850	26 (3.1)	0.96

Notes: The data presented in this table are derived from a questionnaire conducted among participating CURES regional centres, which inquired about the actual bleeding risk scores most commonly used in clinical practice by each centre. Only centres that provided valid questionnaire responses, were tertiary hospitals, and enrolled more than 50 patients with PE were included in the analysis.
[a]The major bleeding rates are based on the CURES data.

 © 2025. The Author(s).

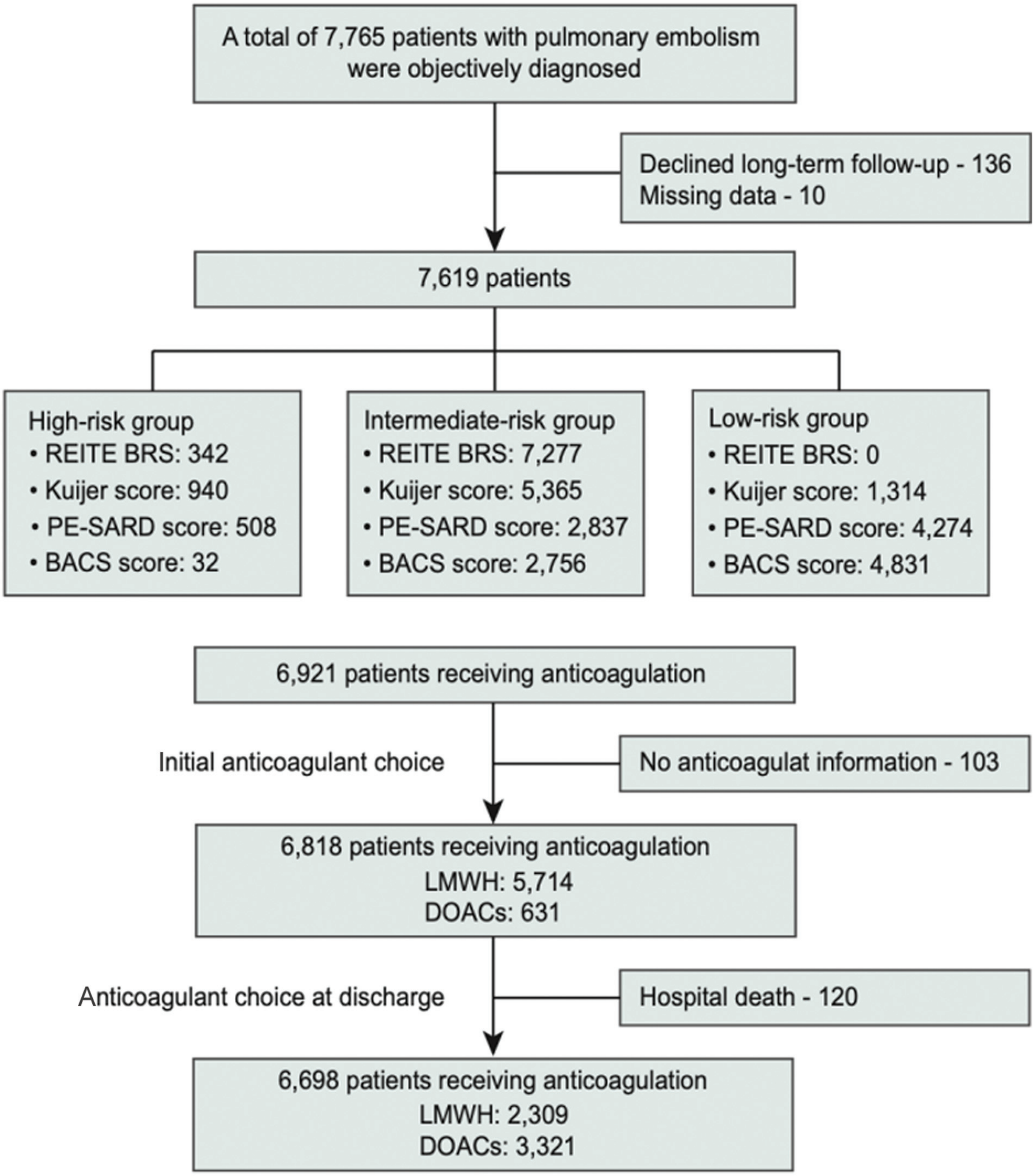

Supplementary Fig. S1 Study cohort flow diagram.

 © 2025. The Author(s).

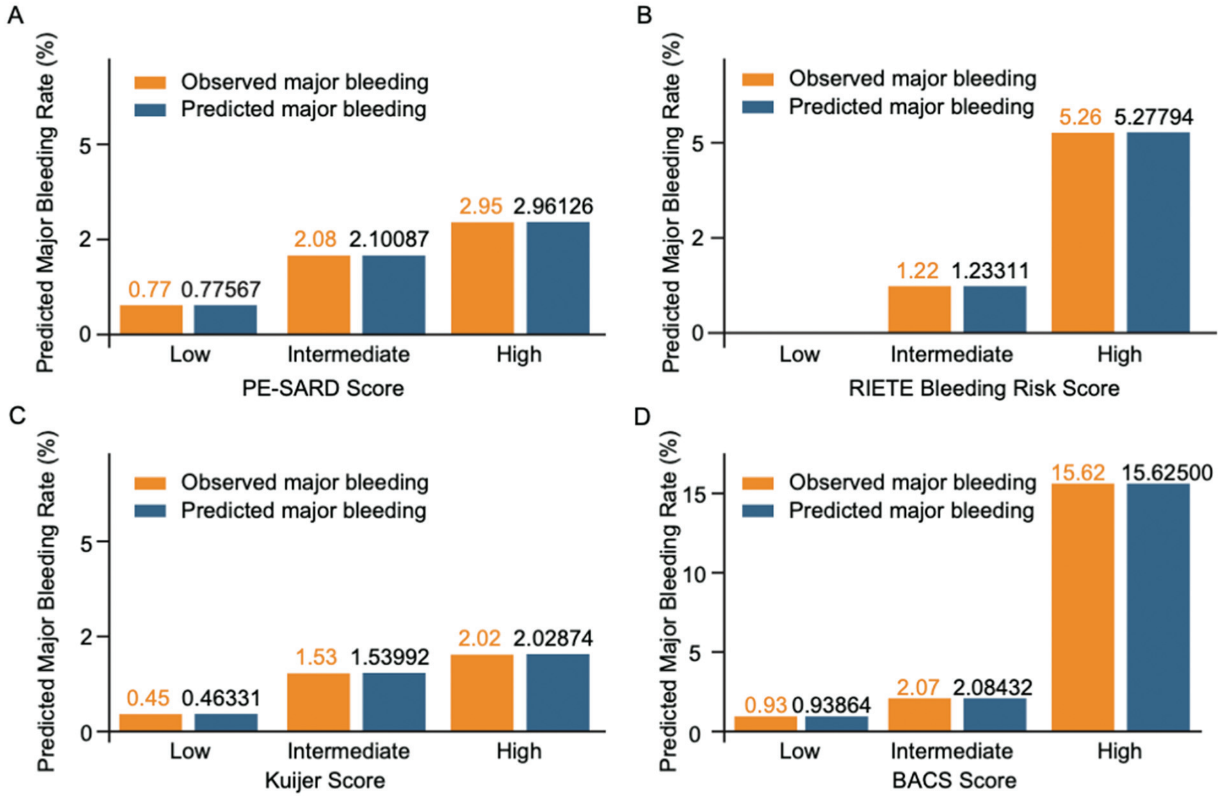

Supplementary Fig. S2 Observed and predicted rates of major bleeding according to bleeding risk classification. (**A**) PE-SARD. (**B**) RIETE. (**C**) Kuijer. (**D**) BACS.

 © 2025. The Author(s).

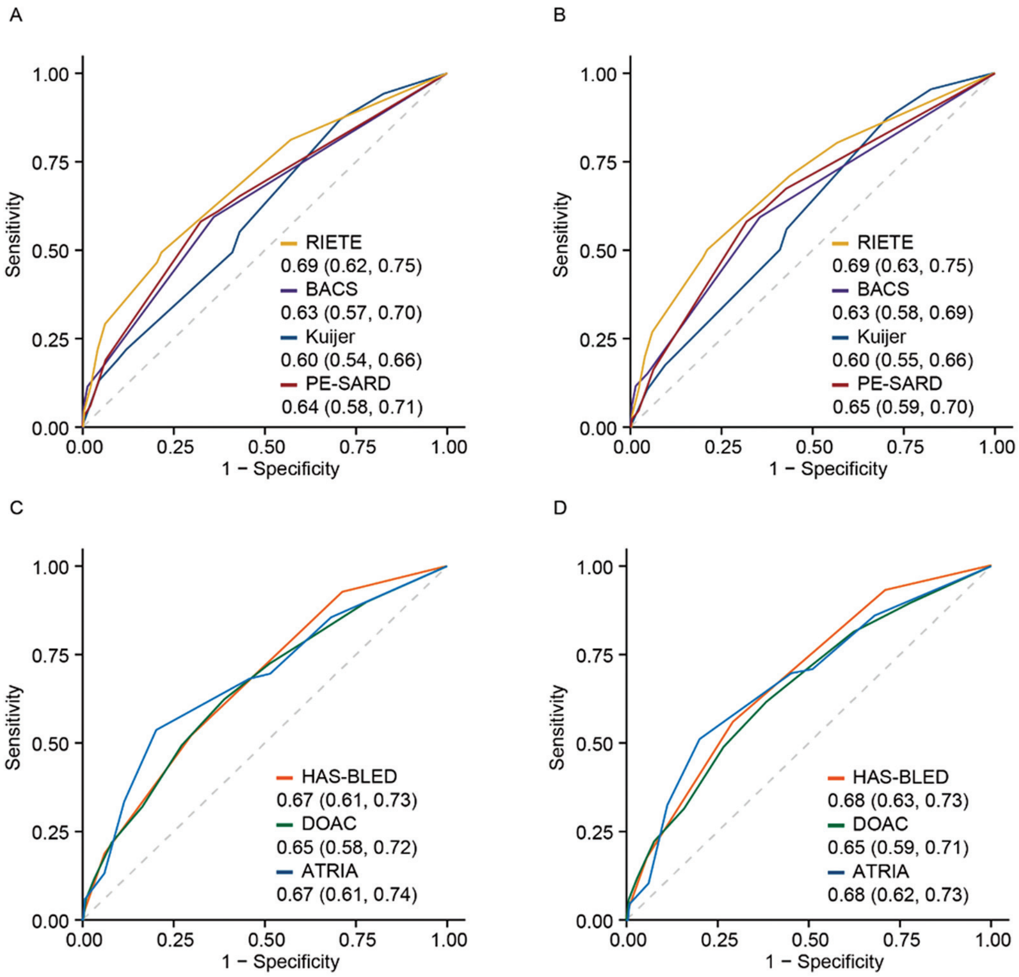

Supplementary Fig. S3 Time-dependent receiver operating characteristic curves for seven bleeding risk scores. (**A**) AUCs for 14-day major bleeding according to RIETE (0.69, 95% CI, 0.62–0.75), BACS (0.63, 95% CI, 0.57–0.70), Kuijer (0.60, 95% CI, 0.54–0.66), and PE-SARD (0.64, 95% CI, 0.58–0.71) scores. (**B**) AUCs for 30-day major bleeding according to RIETE (0.69, 95% CI, 0.63–0.75), BACS (0.63, 95% CI, 0.58–0.69), Kuijer (0.60, 95% CI, 0.55–0.66), and PE-SARD (0.65, 95% CI, 0.59–0.70) scores. (**C**) AUCs for 14-day major bleeding according to HAS-BLED (0.67, 95% CI, 0.61–0.73), DOAC (0.65, 95% CI, 0.58–0.72), and ATRIA (0.67, 95% CI, 0.61–0.74) scores. (**D**) AUCs for 30-day major bleeding according to HAS-BLED (0.68, 95% CI, 0.63–0.73), DOAC (0.65, 95% CI, 0.59–0.71), and ATRIA (0.68, 95% CI, 0.62–0.73) scores. AUC, area under the curve.

 © 2025. The Author(s).

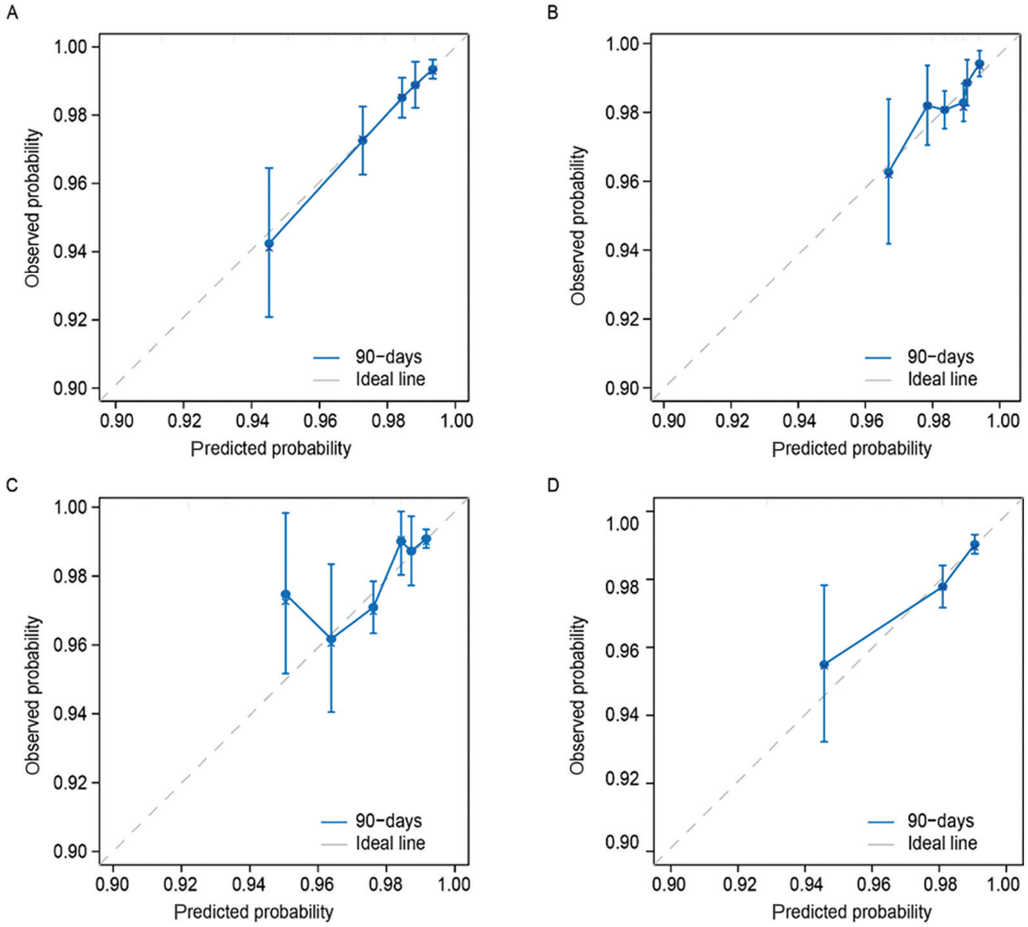

Supplementary Fig. S4 Calibration curves for bleeding risk scores derived from the venous thromboembolism (VTE) population in predicting major bleeding events. (**A**) RIETE. (**B**) Kuijer. (**C**) PE-SARD. (**D**) BACS.

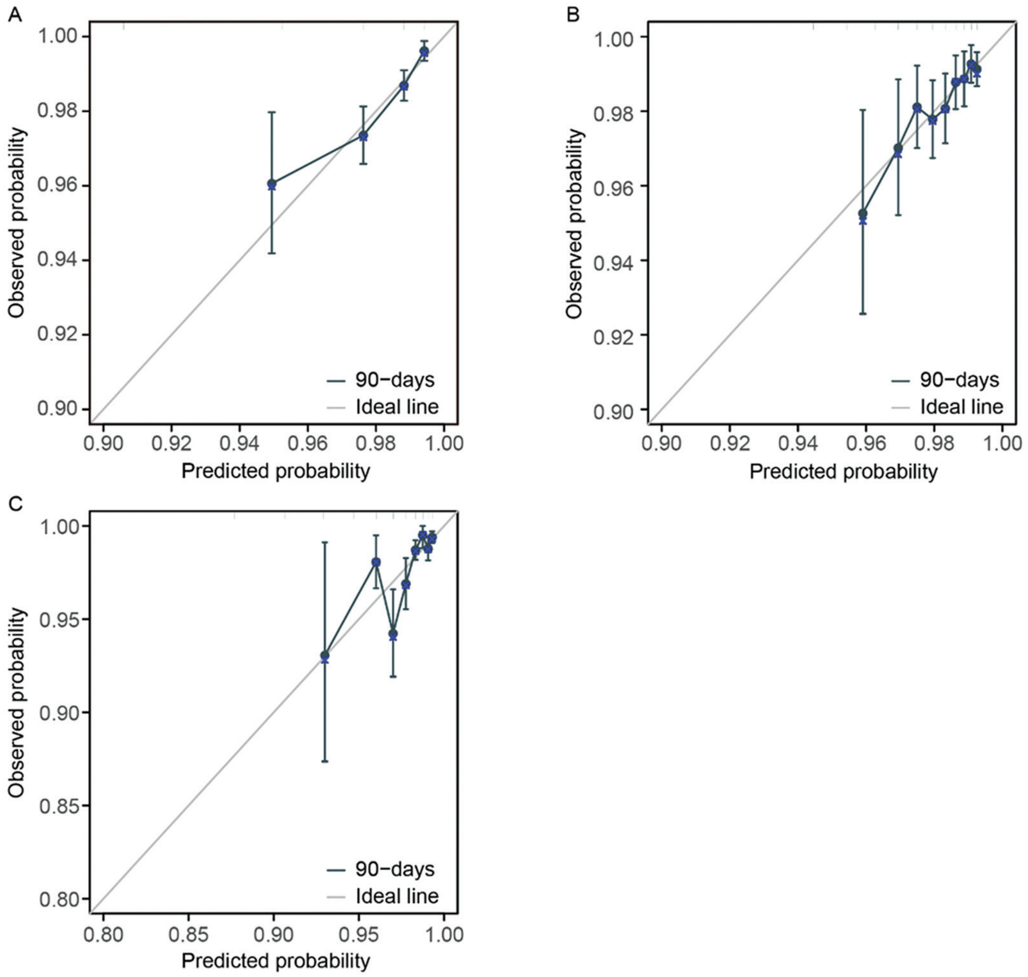

Supplementary Fig. S5 Calibration curves of bleeding risk scores derived from the atrial fibrillation (AF) population for predicting major bleeding events. (**A**) HAS-BLED. (**B**) DOAC. (**C**) ATRIA.

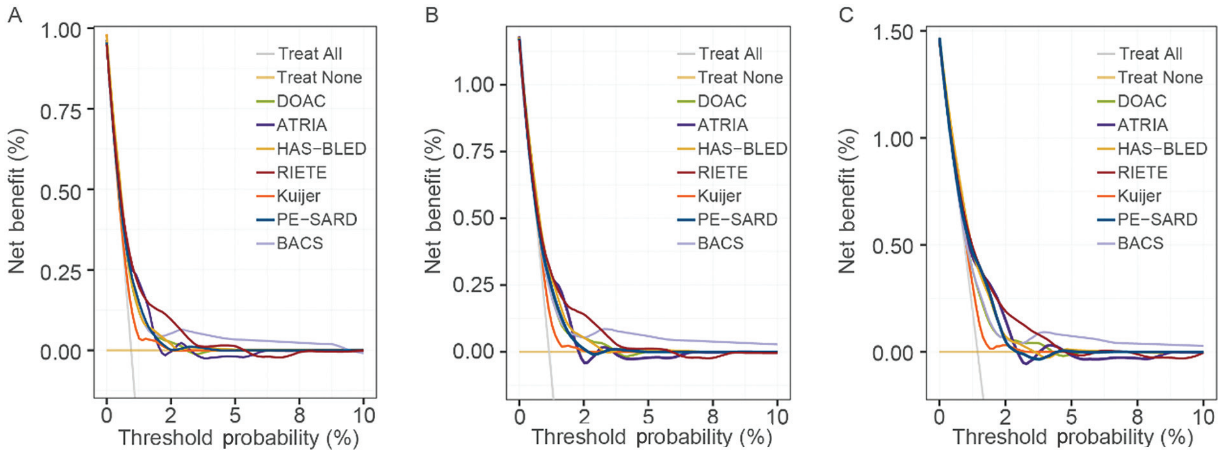

Supplementary Fig. S6 Decision curve analysis for predicting major bleeding events using various bleeding risk scores. (**A**) 14-day major bleeding. (**B**) 30-day major bleeding. (**C**) 90-day major bleeding.

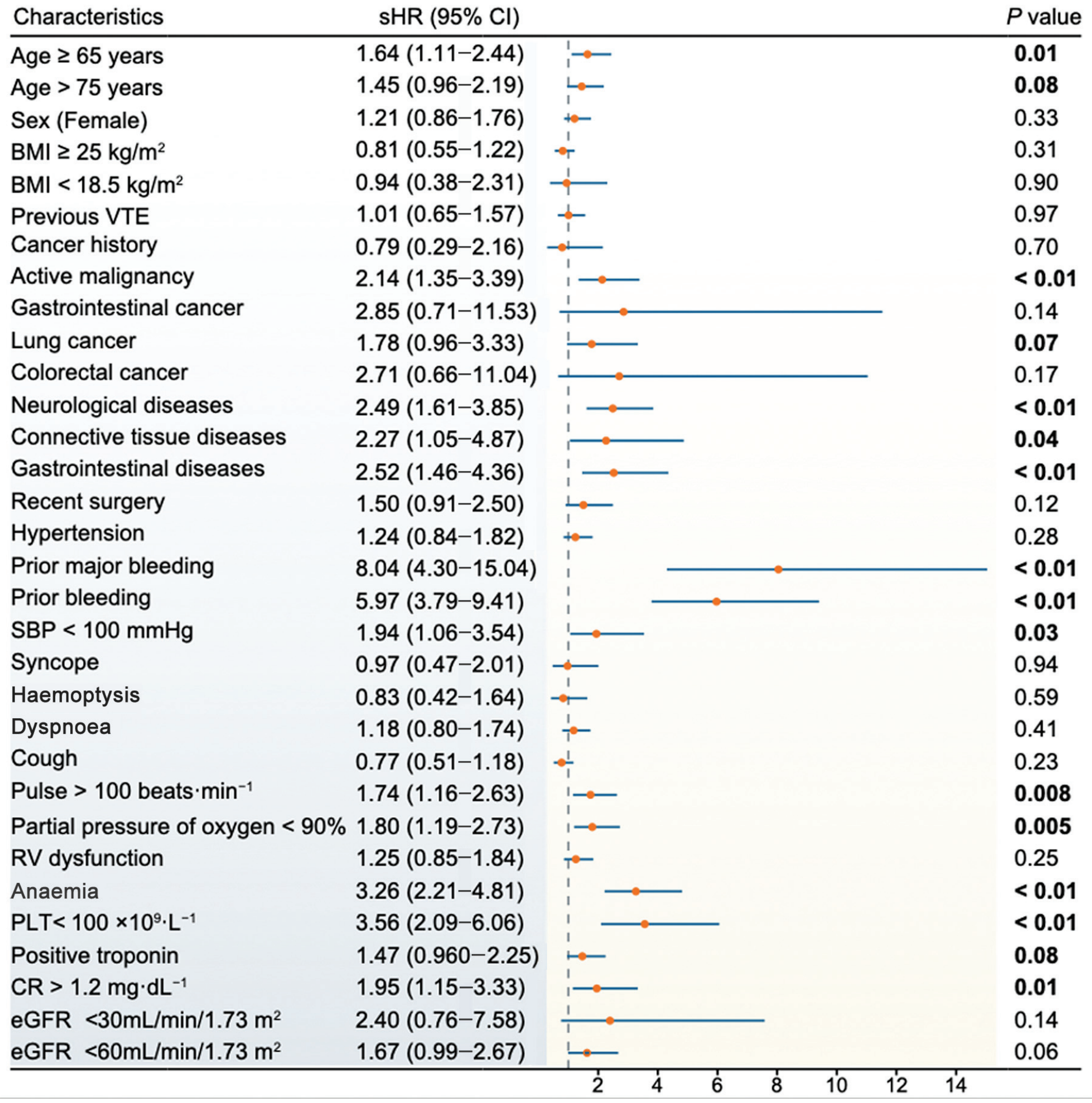

Supplementary Fig. S7 Univariate analysis of 90-day major bleeding in patients with pulmonary embolism. BMI, body mass index; CR, creatinine; eGFR, estimated glomerular filtration rate; PLT, platelet; RV, right ventricle; SBP, systolic blood pressure; VTE, venous thromboembolism.

 © 2025. The Author(s).

Characteristics	sHR (95% CI)	*P* value
Age ≥ 65 years	1.61 (1.06–2.47)	**0.03**
Sex (Female)	1.11 (0.73–1.69)	0.63
Active malignancy	1.68 (1.02–2.74)	**0.04**
Gastrointestinal diseases	2.04 (1.17–3.55)	**0.01**
Hypertension	1.07 (0.70–1.62)	0.77
Prior major bleeding	5.75 (2.97–11.15)	**< 0.01**
Syncope	1.07 (0.52–2.19)	0.85
Pulse > 100 beats·min^{-1}	1.61 (1.07–2.43)	**0.02**
Anaemia	2.49 (1.61–3.83)	**< 0.01**
PLT< 100 ×10^{9}·L^{-1}	2.72 (1.55–4.76)	**< 0.01**
CR > 1.2 mg·dL^{-1}	1.77 (1.02–3.07)	**0.04**

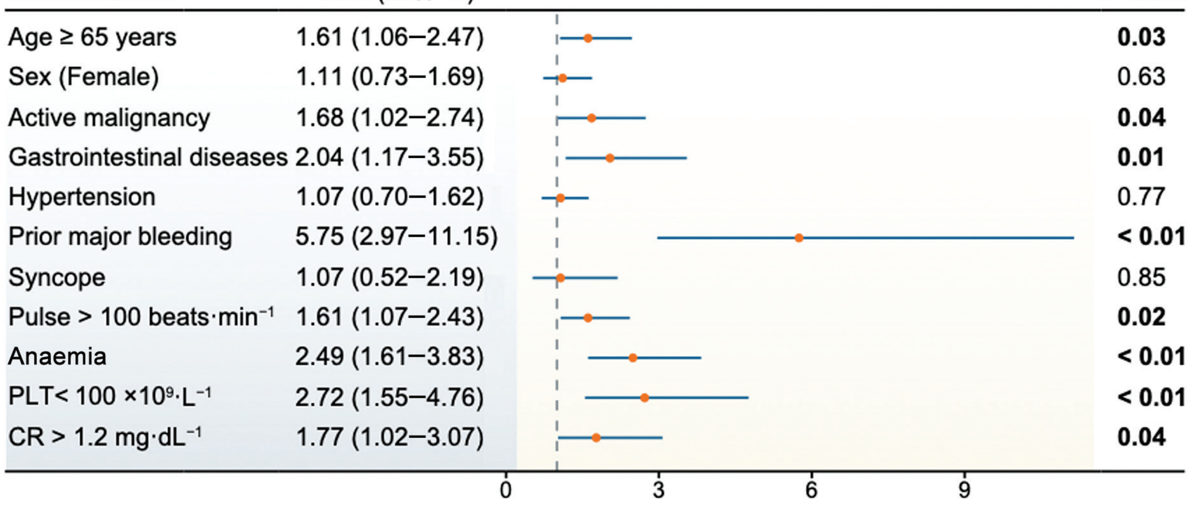

Supplementary Fig. S8 Multivariate analysis of 90-day major bleeding in patients with pulmonary embolism. CR, creatinine; PLT, platelet.

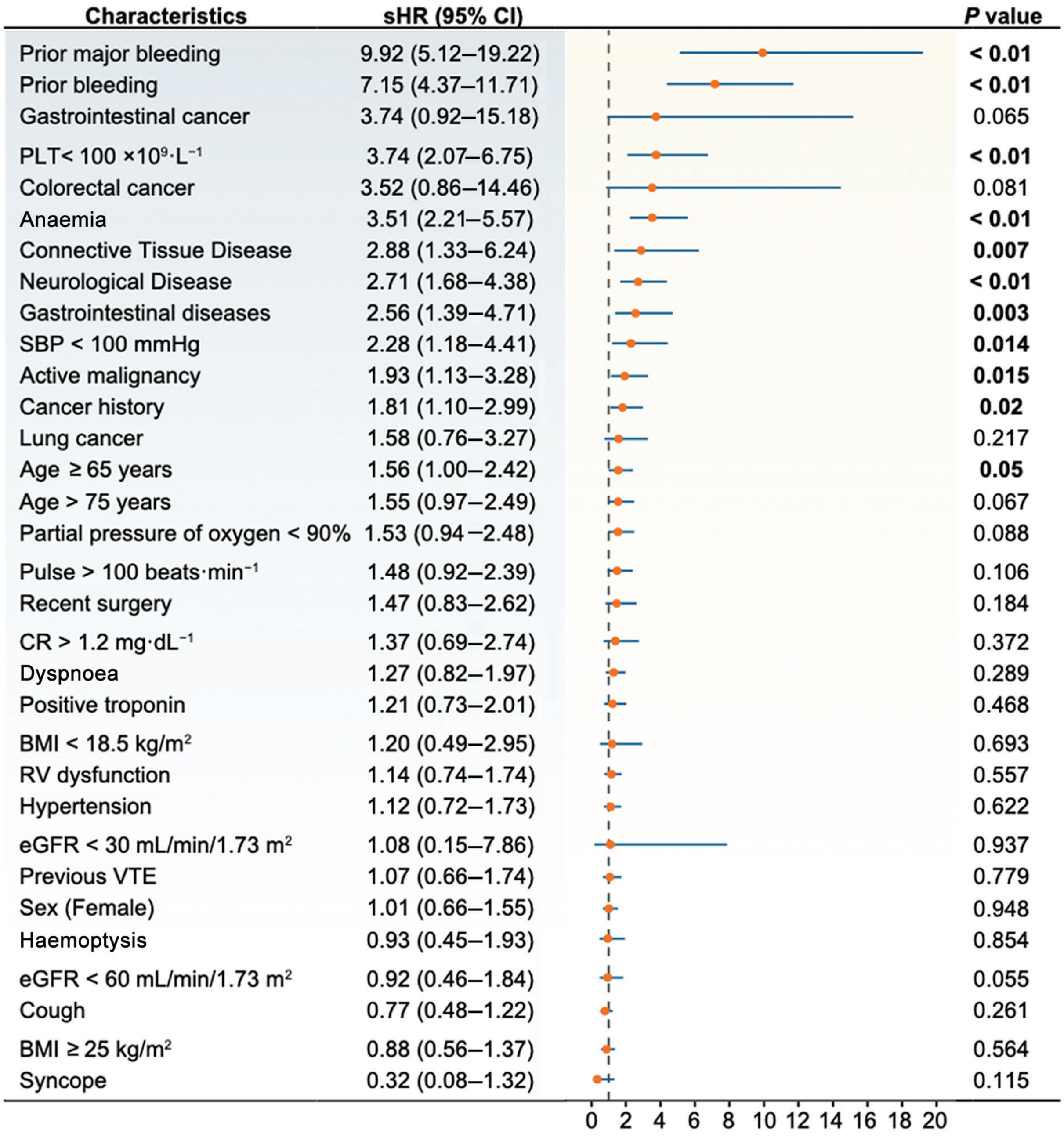

Supplementary Fig. S9 Univariate analysis of 90-day major bleeding in pulmonary embolism patients receiving anticoagulation. BMI, body mass index; CR, creatinine; eGFR, estimated glomerular filtration rate; PLT, platelet; RV, right ventricle; SBP, systolic blood pressure; VTE, venous thromboembolism.

 © 2025. The Author(s).

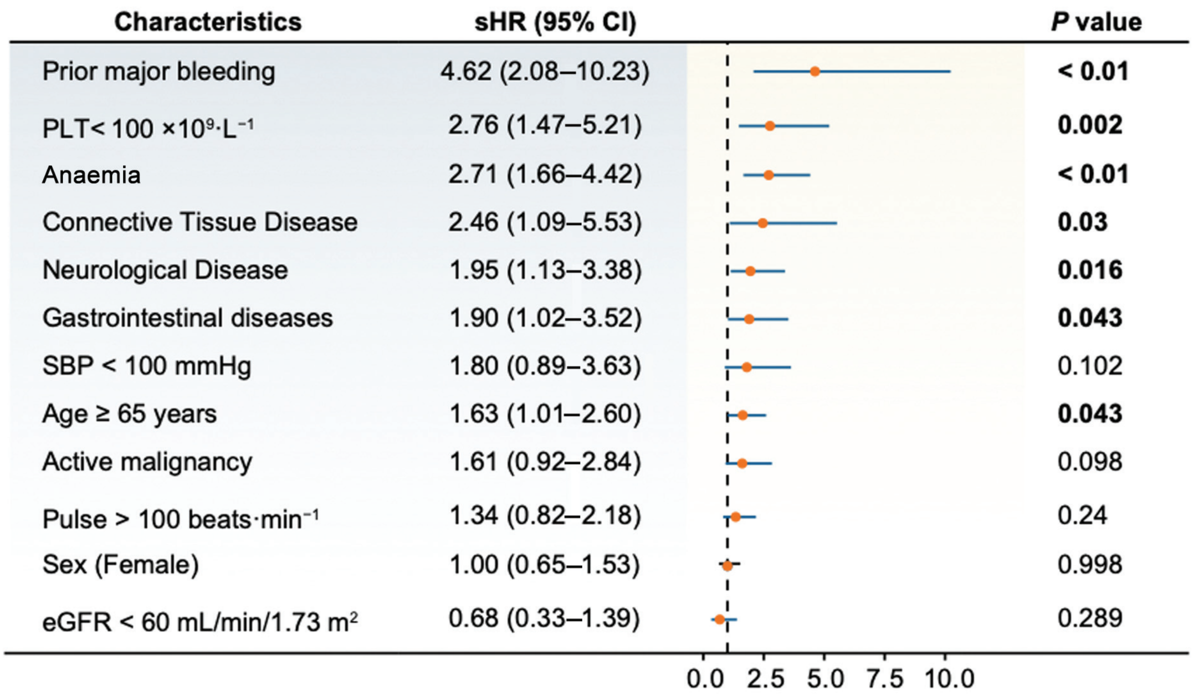

Supplementary Fig. S10 Multivariate analysis of 90-day major bleeding in pulmonary embolism patients receiving anticoagulation. eGFR, estimated glomerular filtration rate; PLT, platelet; SBP, systolic blood pressure.

 © 2025. The Author(s).

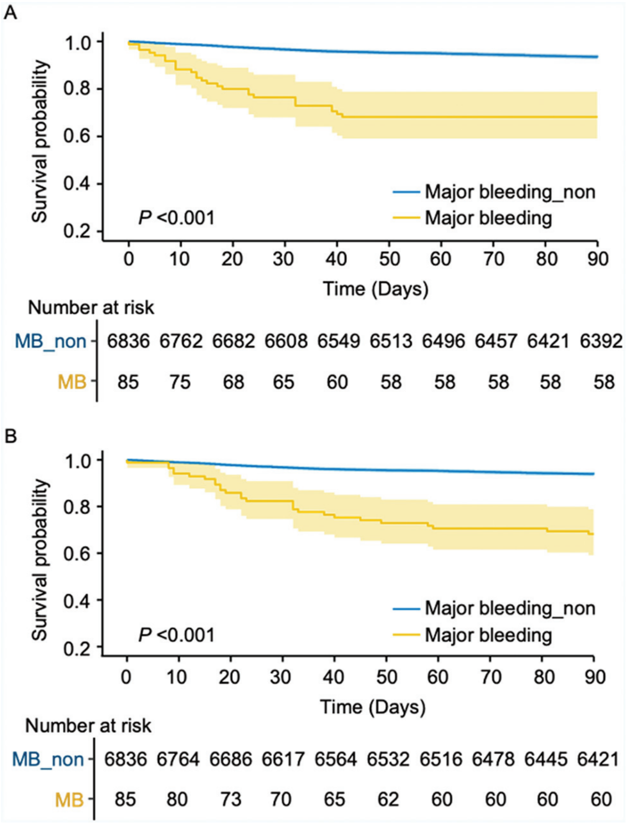

Supplementary Fig. S11 Impact of major bleeding (MB) on outcomes in anticoagulated patients with pulmonary embolism. (**A**) Kaplan-Meier curve for 90-day composite outcomes (including VTE recurrence and all-cause mortality). (**B**) Kaplan-Meier curve for 90-day mortality.

 © 2025. The Author(s).

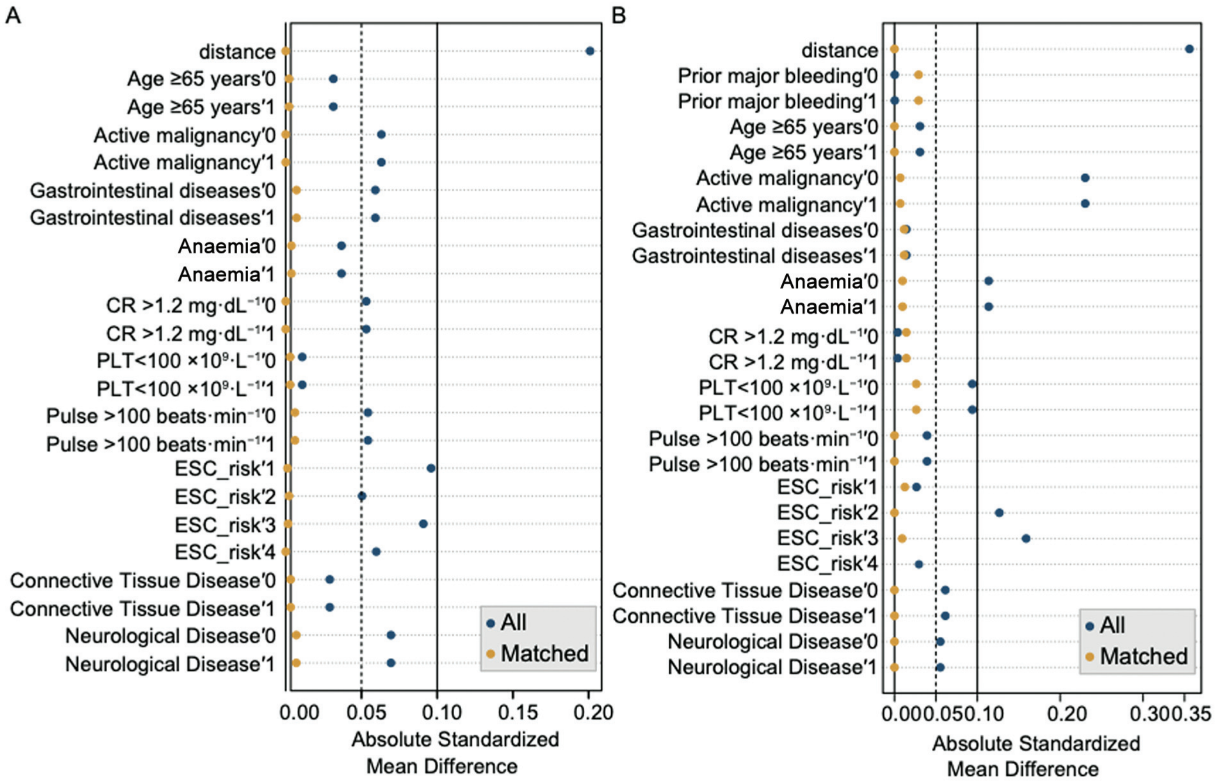

Supplementary Fig. S12 Assessing the quality of matches between DOAC and LMWH groups at discharge. (**A**) Standardized mean difference (SMD) before and after propensity score matching (PSM) in patients classified as low bleeding risk according to the RIETE BRS. (**B**) SMD before and after PSM in patients classified as high bleeding risk according to the RIETE BRS. CR, creatinine; ESC, European Society of Cardiology; PLT, platelet.

 © 2025. The Author(s).

References

1 Schulman S, Kearon CSubcommittee on Control of Anticoagulation of the Scientific and Standardization Committee of the International Society on Thrombosis and Haemostasis. Definition of major bleeding in clinical investigations of antihemostatic medicinal products in non-surgical patients. J Thromb Haemost 2005;3(04):692–694

2 Kaatz S, Ahmad D, Spyropoulos AC, Schulman SSubcommittee on Control of Anticoagulation. Definition of clinically relevant non-major bleeding in studies of anticoagulants in atrial fibrillation and venous thromboembolic disease in non-surgical patients: communication from the SSC of the ISTH. J Thromb Haemost 2015;13(11):2119–2126

3 Büller HR, Décousus H, Grosso MA, et al; Hokusai-VTE Investigators. Edoxaban versus warfarin for the treatment of symptomatic venous thromboembolism. N Engl J Med 2013;369(15):1406–1415

4 Ruíz-Giménez N, Suárez C, González R, et al; RIETE Investigators Findings from the RIETE Registry. Predictive variables for major bleeding events in patients presenting with documented acute venous thromboembolism. Thromb Haemost 2008;100(01): 26–31

5 Kuijer PM, Hutten BA, Prins MH, Büller HR. Prediction of the risk of bleeding during anticoagulant treatment for venous thromboembolism. Arch Intern Med 1999;159(05):457–460

6 Chopard R, Piazza G, Falvo N, et al. An original risk score to predict early major bleeding in acute pulmonary embolism: the Syncope, Anemia, Renal Dysfunction (PE-SARD) bleeding score. Chest 2021;160(05):1832–1843

7 Jara-Palomares L, Jiménez D, Bikdeli B, et al; RIETE investigators RIETE Steering Committee Members: Members of the RIETE Group. Derivation and validation of a clinical prediction rule for thrombolysis-associated major bleeding in patients with acute pulmonary embolism: the BACS score. Eur Respir J 2020;56(06): 2002336

 © 2025. The Author(s).
